# Species Tree Branch Length Estimation despite Incomplete Lineage Sorting, Duplication, and Loss

**DOI:** 10.1093/gbe/evaf200

**Published:** 2025-11-26

**Authors:** Yasamin Tabatabaee, Chao Zhang, Shayesteh Arasti, Siavash Mirarab

**Affiliations:** Department of Computer Science, University of Illinois at Urbana-Champaign, Urbana, IL, USA; GLOBE Institute, University of Copenhagen, Copenhagen, Denmark; Department of Computer Science and Engineering, University of California, San Diego, CA, USA; Department of Electrical and Computer Engineering, University of California San Diego, San Diego, CA, USA

**Keywords:** phylogenomics, species trees, branch length estimation, incomplete lineage sorting, gene duplication and loss, horizontal gene transfer

## Abstract

Phylogenetic branch lengths are essential for many analyses, such as estimating divergence times, analyzing rate changes, and studying adaptation. However, true gene tree heterogeneity due to incomplete lineage sorting, gene duplication and loss, and horizontal gene transfer can complicate the estimation of species tree branch lengths. While several tools exist for estimating the topology of a species tree addressing various causes of gene tree discordance, much less attention has been paid to branch length estimation on multi-locus datasets. For single-copy gene trees, some methods are available that summarize gene tree branch lengths onto a species tree, including coalescent-based methods that account for heterogeneity due to incomplete lineage sorting. However, no such branch length estimation method exists for multi-copy gene family trees that have evolved with gene duplication and loss. To address this gap, we introduce the CASTLES-Pro algorithm for estimating species tree branch lengths while accounting for both gene duplication and loss and incomplete lineage sorting. CASTLES-Pro improves on the existing coalescent-based branch length estimation method CASTLES by increasing its accuracy for single-copy gene trees and extending it to handle multi-copy ones. Our simulation studies show that CASTLES-Pro is generally more accurate than alternatives, eliminating the systematic bias toward overestimating terminal branch lengths often observed when using concatenation. Moreover, while not theoretically designed for horizontal gene transfer, we show that CASTLES-Pro is relatively robust to random horizontal gene transfer, though its accuracy can degrade at the highest levels of horizontal gene transfer.

Significance A scalable approach to species tree estimation is the two-step method, which involves first computing the gene trees and then summarizing them. Many methods exist for summarizing the topologies of the gene trees, but less attention has been paid to the branch lengths. In this paper, we introduce the CASTLES-Pro method to estimate species tree branch lengths in units of substitutions per site, giving a method that can handle incomplete lineage sorting and duplication and loss, a feature that our previous method, CASTLES, lacked. CASTLES-Pro improves the accuracy of branch lengths for single-copy genes and enables branch length estimation from multi-copy gene trees.

## Introduction

Summarizing a collection of potentially conflicting trees inferred from different parts of the genome (i.e. gene trees) to obtain a species tree has now become a routine analysis. This approach promises to account for biological processes such as incomplete lineage sorting (ILS), gene duplication and loss (GDL), and horizontal gene transfer (HGT) that create discordance between gene trees and the species tree (Maddison 1997). Prior studies have confirmed that the most accurate methods for estimating the topology of species trees are those that take biological sources of heterogeneity into account (Kubatko and Degnan 2007; Molloy and Warnow 2018; Jiang et al. 2020). Several methods use likelihood under a model of genome evolution coupled with Bayesian MCMC inference to jointly estimate both topology and branch lengths of gene trees and species trees (Liu 2008; Ogilvie et al. 2017; Flouri et al. 2018). These methods tend to be accurate, but they are computationally intensive. An alternative approach is to infer gene trees independently and then use a summary method to construct a species tree. This 2-step approach has been more scalable and generally accurate (Mirarab et al. 2021), spurring the development of many such methods (e.g. Liu et al. 2009; Larget et al. 2010; Liu and Yu 2011; Vachaspati and Warnow 2015; Solís-Lemus and Ané 2016; Wang and Nakhleh 2018). Some of these summary methods (e.g. Wehe et al. 2008; Chaudhary et al. 2013; Molloy and Warnow 2020; Zhang et al. 2020; Legried et al. 2021; Willson et al. 2022) account for GDL and can take as input multi-copy gene trees, vastly expanding the set of loci that can be used (Smith and Hahn 2021). For example, the ASTRAL-like methods use variants of the median tree problem based on the quartet distance (Mirarab et al. 2014; Zhang et al. 2025) and have been extended to multi-copy input trees (Zhang et al. 2020). The ASTRAL family is widely used, including the ASTRAL-Pro extension to multi-copy input (e.g. Guo et al. 2021; Chanderbali et al. 2022; Ding et al. 2023; Li et al. 2024).

Species trees are most useful if they are furnished with branch lengths, as many downstream applications, including dating, comparative genomics, and the study of diversification and adaptation, depend on branch lengths. However, widely used summary methods such as ASTRAL do not produce the branch lengths needed for downstream analysis. Species trees can be furnished with coalescent unit (CU) branch length (Sayyari and Mirarab 2016), but these are only available for internal branches (unless multiple individuals are available per species), and GDL-based methods typically can only infer gene birth/death rates for each branch. Meanwhile, downstream applications often require branch lengths in the unit of either substitution per site (SU) or time. The standard *ad-hoc* solution is to estimate the species tree topology using a summary method and then infer the branch lengths using concatenation. Often, branch lengths are optimized on a fixed topology using maximum likelihood applied to a concatenated sequence of all genes (e.g. Song et al. 2012; Jarvis et al. 2014; Zhu et al. 2019). An alternative is using distance-based approaches, such as ERaBLE (Binet et al. 2016) and TCMM (Arasti et al. 2024), to summarize patristic distances from gene trees onto the species tree. While distance-based methods use gene trees as input, just like concatenation, they do not directly model the biological processes that create gene tree heterogeneity, reducing their theoretical justification. Nevertheless, these methods have the potential advantage of being agnostic to the source of discordance, while concatenation has the advantage of not relying on accurate gene tree estimation. Ultimately, which approach should be preferred is an empirical question with important downstream implications (Moody et al. 2022).

There are also methods specifically designed for estimating speciation times under the multi-species coalescent (MSC) model. For instance, Peng et al. (2022) proposed a maximum a posteriori (MAP) estimator based on composite likelihood for inferring speciation times under the MSC model combined with the JC69 (Jukes and Cantor 1969) DNA substitution model assuming a strict molecular clock. Kubatko et al. (2024) established that for species trees with three and four taxa, the speciation times are identifiable under the MSC+JC69 model with a strict molecular clock and derived an estimator for species tree branch lengths based on site pattern probabilities. However, the reliance of these methods on the strict molecular clock assumption and the computational challenges of extending them to larger trees limit their applicability to large datasets with branch rate heterogeneity.

We recently introduced the CASTLES (Tabatabaee et al. 2023) method for estimating SU branch length for a fixed species tree topology, specifically designed to handle ILS, as modeled by the MSC. CASTLES estimates species divergence times as opposed to *genic* divergences, which are expected to be older (Edwards and Beerli 2000). CASTLES had higher accuracy than alternatives in our simulations. Nevertheless, it has several limitations. Most importantly, CASTLES is limited to single-copy gene trees and, therefore, cannot be used with multi-copy input trees, severely limiting its applicability. To our knowledge, the only method that can estimate SU branch lengths from multi-copy gene trees is SpeciesRax (Morel et al. 2022), which does model GDL but does not model ILS and, thus, deep coalescence. In addition, the study by Willson et al. (2022) shows that SpeciesRax can be less accurate than ASTRAL-Pro and other methods in conditions with ILS and is also less scalable. Even the use of concatenation in the presence of GDL is complicated and requires additional techniques, such as DISCO (Willson et al. 2022), to decompose multi-copy genes into single-copy ones. Beyond the lack of support for GDL, CASTLES has not previously been tested under conditions with HGT, and even for ILS, it required several approximations that could reduce its accuracy.

In this article, we advance the CASTLES methodology to address its major limitations and broaden the scope of conditions under which it is tested. We present a dynamic programming algorithm for estimating branch lengths of a species tree from multi-copy gene family trees that have evolved with GDL in addition to ILS, leading to a new method called CASTLES-Pro. In addition, we improve upon CASTLES by relaxing some approximations and modifying other assumptions. Beyond ILS and GDL, for which it is designed, we use simulations to test how CASTLES-Pro performs under conditions that include substantial levels of ILS and HGT. In simulations, we show that the method is accurate, robust to various sources of heterogeneity, and scalable to thousands of species and genes. On diverse biological data ranging from the root of the tree of life to recent speciations, we show that using CASLTES-Pro instead of concatenation dramatically alters branch lengths. We have incorporated CASTLES-Pro inside the ASTER package of tools (Zhang et al. 2025), providing a new C++ implementation compared to CASTLES. Thus, any user of ASTRAL-Pro or ASTRAL-IV would automatically obtain SU branch lengths with no additional step needed.

## Results

We compare CASTLES-Pro to other branch length estimation methods using three sets of simulated datasets and nine published biological datasets with gene tree discordance due to ILS, GDL, and HGT (Table 1). We compare CASTLES-Pro to CASTLES, ERaBLE, FastME (Lefort et al. 2015) used on matrices of average patristic distances (referred to as FASTME(AVG)), and concatenation using maximum likelihood with RAxML (Stamatakis 2014).

**Table 1.** Statistics of the simulated and biological datasets used in this study.

Simulated	*n*	*k*	${\left\|S-G\right\|}^{§}$	$\left\|G-\hat{G}\right\|$	Gene len. (bp)	See
ILS-only	101	1,000	30%–58%	23, 31, 42, 55%	1,600, 800, 400, 200	
ILS+GDL	21–1,001	50–10,000	15%–78%*	18% – 56%	50, 100, 500	Table S4
ILS+HGT	51	1,000	30%–68%	28%	1,000	Table S5

Biological	*n*	*k*	Type	$\left\|\hat{S}-\hat{G}\right\|$ ^¶^	Total len. (bp)^#^	See
Birds	363	63,430	ILS	31.1%	63,430,000	Table S6
Bees	32	853	ILS	39.7%	576,041	Table S6
Mammals	37	424	ILS	26.6%	1,385,220	Table S6
Fungi	16	706^†^, 7,180^‡^	ILS+GDL	NA	286,114	Table S6
Plants(1kp)	80	424^†^, 9,610^‡^	ILS+GDL	48.0%	290,718	Table S6
Eudicots	40	345^†^, 2,573^‡^	ILS+GDL	50.5%	262,343	Table S6
AB core	72	49	HGT	69.3%	10,522	Table S6
AB non-rib.	108	38	HGT	61.8%	6,534	Table S6
AB WoL	10,575	381	HGT	53.9%	1,162,421,084	Table S6

§ Average discordance between two types of trees is denoted by ‖a−b‖ and is measured using the average RF distance. *S* and $\hat{S}$ denote model and estimated species trees; *G* and $\hat{G}$ denote true and estimated gene trees.

* RF between true gene trees and the true locus tree, which is only due to ILS.

† single-copy genes, used in concatenation.

‡ multi-copy genes used for ASTRAL-Pro and CASTLES-Pro.

¶ RF distance between single-copy gene trees and species trees estimated by ASTRAL or ASTRAL-Pro (NA when not available).

# The total length of the sequence alignments for the single-copy loci.

*n* denotes the number of species and *k* denotes the number of single-copy or multi-copy genes.

The simulations are all using the Simphy (Mallo et al. 2016) simulator, with modifications we made to output true branch lengths in the units of substitutions per site, defined as the length in generation time multiplied by the substitution rate of the branch (both already present in Simphy). All the datasets we use are adopted from previous analyses. In all simulations, we estimate branch lengths on the fixed true species tree topology. We measure branch length estimation error using three metrics: mean absolute error (MAE) ($\left|\bar{t}-t\right|$), mean logarithmic error ($\left|\log\;\bar{t}-\log\;t\right|$), and the bias of the estimated length $\bar{t}-t$ (*t* and $\bar{t}$ are true and estimated branch lengths, resp.) averaged across all species tree branches. The log error emphasizes short branches, while the MAE and bias emphasize long branches. We remove the outgroup before measuring the branch length estimation error. For all methods, we replace negative and zero branch lengths with a small pseudo-count ($10^{-6}$) before calculating error metrics.

### ILS-only Simulations

**Fig. 1.**
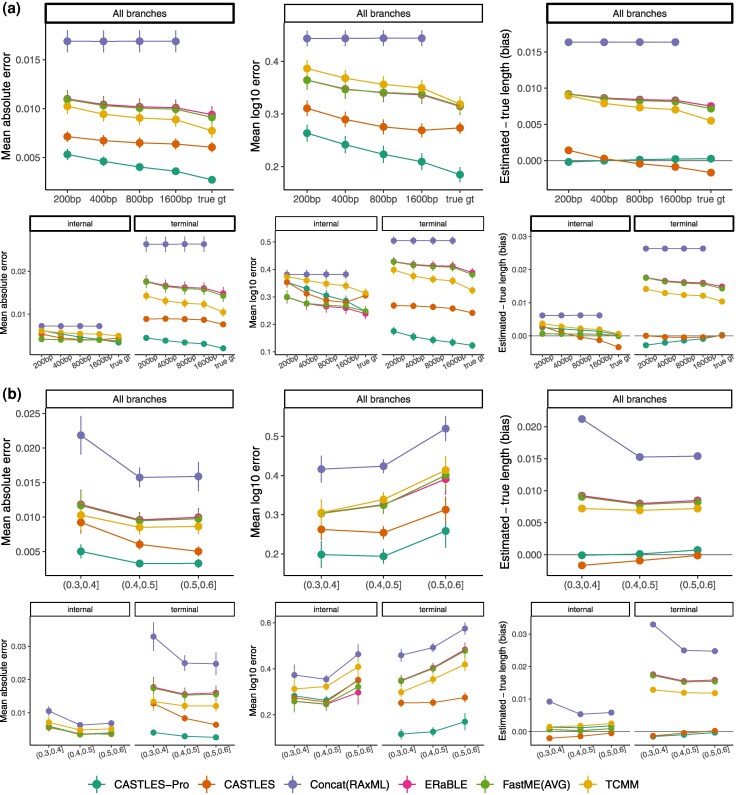
Mean absolute error (MAE), mean log error, and bias for different branch length estimation methods on 100-taxon simulated ILS datasets. The average discordance (AD) due to ILS level on this dataset is 47%. The number of genes is 1,000, and the number of replicates is 50. a) Varying gene tree error (GTEE) (*x*-axis) where the GTEE level changes between 0% for true gene trees to 55% for gene trees estimated from 200 bp alignments. b) Varying the level of ILS (*x*-axis) for conditions with 1,600 bp sequence length. The number of replicates in the three ILS bins are 9, 29, and 12, respectively. See also Fig. S6 for comparison between CASTLES-Pro and CASTLES-Pro+TCMM.

We start by examining an ILS-only 100-taxon dataset adopted from Zhang et al. (2018) with heterogeneous levels of ILS (controlled by tree height) and levels of Gene Tree Estimation Error (GTEE) controlled by changing sequence length (Table 1). CASTLES-Pro has the best accuracy across all GTEE levels of this dataset, followed by CASTLES (Fig. 1 and Fig. S6). Distance-based methods come next, with TCMM outperforming ERaBLE and FastME(AVG), and concatenation is the least accurate overall. Relative accuracy of methods is mostly consistent across different alignment lengths (Fig. 1a) and levels of ILS (Fig. 1b). As the amount of ILS increases, MAE decreases for all methods, likely due to the reduced tree height (and thus branch lengths), while log error increases because it focuses on relative error. As alignment length increases (and GTEE decreases), concatenation remains stable while CASTLES-Pro and distance-based methods become successively better. Improvements of CASTLES-Pro are mostly due to better terminal branches, which are substantially more accurate than the other methods in all conditions (Fig. 1). On internal branches, the relative accuracy depends on the condition. CASTLES-Pro is better for true gene trees and slightly worse than the distance-based methods and CASTLES for the highest GTEE level. Finally, note that pairing TCMM with CASTLES-Pro, as described by Arasti et al. (2024), substantially reduces the error of TCMM, but in these ILS-only conditions, the combination is not as good as CASTLES-Pro alone (Fig. S6).

In addition to better accuracy, CASTLES-Pro has the lowest overall bias (Fig. 1 and Fig. S6). In particular, concatenation has a substantial overestimation bias for terminal branches, but it also overestimates internal branches to a lesser degree. CASTLES shifts from a slight underestimation bias for true gene trees to a minor overestimation bias for gene trees with high GTEE; this is due to the effects of the imprecise Lambert approximation used in CASTLES, which is fixed in CASTLES-Pro (see Materials and Methods). Distance-based methods also exhibit an overestimation bias, though it is smaller than that of concatenation. Evaluating terminal and internal branches separately (Fig. 1) shows that CASTLES-Pro is unbiased for both terminal and internal branches when given true gene trees; as the GTEE increases, it suffers a small overestimation bias for internal branches and a similar underestimation bias for terminal ones; thus, effects of gene tree error are reduced but not fully eliminated in CASTLES-Pro. Finally, distance-based methods have a small overestimation bias for internal branches that increases as GTEE increases, and a much larger bias for terminal branches.

The trends for log error, which emphasizes short branches more than MAE, are similar, and CASTLES-Pro is the most accurate method overall, followed by CASTLES, distance-based methods, and finally concatenation. The only difference, according to the log error, is that TCMM is overall slightly less accurate than the other two distance-based methods (ERaBLE and FastME(AVG)), but still more accurate than them for terminal branches.

### GDL+ILS Simulations

We next examined simulations with both GDL and ILS, adopted from a dataset by Willson et al. (2022, 2023), including varying numbers of genes, species, and gene tree estimation error (Table 1 and Table S4). Since all existing methods are only designed to work with single-copy genes, to enable comparisons, we added a preprocessing step using the method DISCO (Willson et al. 2022) to decompose gene family trees into single-copy gene trees, which can then be used with various methods, with several caveats (see Material and Methods).

**Fig. 2.**
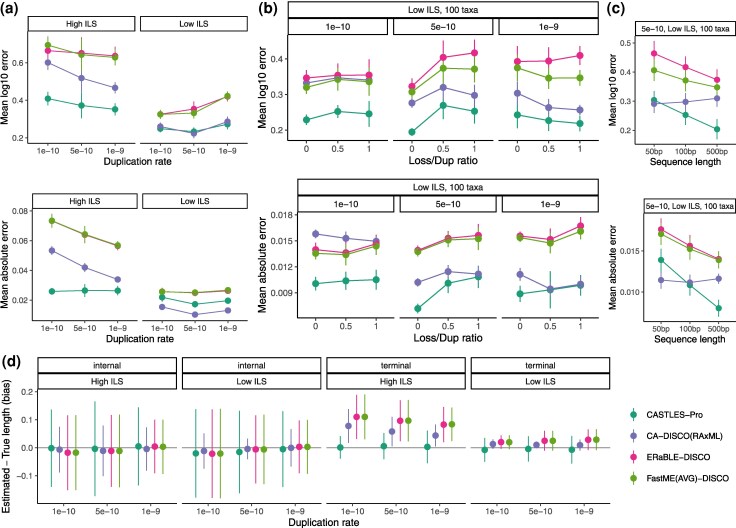
Mean log error, MAE, and bias of branch lengths for simulated GDL+ILS datasets, varying number of species, duplication rate, ILS level, and sequence length. When not specified, each parameter is set to default: 1,000 genes, 100 bp sequence length, equal loss and duplication rates, 20 species. a) 20-taxon datasets varying the duplication rate and ILS rate. b) 100-taxon datasets, low ILS condition, varying duplication rate, and loss/dup ratio. c) 100-taxon datasets, 5e-10 duplication rate, low ILS condition, varying sequence length. d) Bias divided by terminal and internal length for the conditions in panel A. The number of replicates is 10. See Figs. S7–S12 for full results.

On this ILS+GDL dataset, CASTLES-Pro has the lowest error and bias in many but not all conditions, with concatenation used with DISCO (CA-DISCO) performing better with low ILS according to some metrics (Fig. 2). CASTLES-Pro is always the most accurate method in the high ILS conditions, followed by CA-DISCO, but the gap is particularly large for lower duplication rates (Fig. S7) or large numbers of gene trees (Figs. S8 and S9). For the low ILS condition, CASTLES-Pro is still better than CA-DISCO according to log error in most conditions but is outperformed in some conditions according to the MAE metric; in particular, CA-DISCO clearly outperforms CASTLES-Pro with 20 species according to the MAE metric regardless of the duplication rates (Fig. S7), number of genes (Fig. S8), or sequence length (Fig. S10). However, with more species, CASTLES-Pro either outperforms or matches CA-DISCO even with low ILS (Fig. 2 and Fig. S11). Since log error emphasizes short branches more than MAE, these trends suggest that CASTLES-Pro is doing a consistently better job at estimating short branches, whereas concatenation is sometimes better at estimating long branches. Other methods are less competitive. CASTLES run on DISCO decomposed gene trees is less accurate than CASTLES-Pro in most conditions across both 20-taxon and 100-taxon datasets (Figs. S7 to S12). Distance-based methods are the least accurate in almost all conditions.

The number of genes, sites per gene, and species all impact accuracy in various ways. As the number of genes increases, the error of CASTLES-Pro drops faster than CA-DISCO for both 20 (Fig. S8) and 100 species (Fig. S9). Similarly, increasing the sequence length and thus decreasing GTEE does not help CA-DISCO, but makes CASTLES-Pro and other methods more accurate (Fig. S10), with CASTLES-Pro improving the fastest, especially with 100 species (Fig. 2c). The number of species has a mixed impact, which depends on the rate of duplication, the measure of error, and the choice of method (note that tree heights are fixed when the number of species changes, creating shorter branches with more species). The impact of the duplication rate also depends on the level of ILS and the method. Overall, CASTLES-Pro is relatively robust, retaining similar error and bias levels across different duplication rates (Fig. 2 and Fig. S7). Increasing loss rates, however, can increase the error of CASTLES-Pro in some cases but does not introduce any discernible bias (Fig. 2 and Fig. S12). Methods that rely on DISCO to decompose the trees tend to become better with higher duplication rates, especially with high ILS.

Overall, CASTLES-Pro has a lower bias than other methods, especially for terminal branches (Fig. 2d, Figs. S7 to S12). CA-DISCO clearly overestimates terminal branches, especially for higher ILS levels; in contrast, CASTLES-Pro does not have a clear bias for terminal branches. For internal branches, all methods are less biased, with CA-DISCO and CASTLES-Pro performing slightly better for low and high ILS conditions, respectively. The distance-based methods also have a clear overestimation bias for terminal branches. Overall, the most glaring form of bias is for terminal branches for high ILS conditions in all experiments, a problem that CASTLES-Pro eliminates.

### HGT+ILS Simulations

While CASTLES-Pro does not have any theoretical guarantees under HGT, we can hope that for random forms of HGT, its averaging method remains robust. We examined this hypothesis in ILS+HGT simulations, which we recreated based on a dataset by Davidson et al. (2015) with six levels of HGT rates (Table 1). The first four model conditions of this dataset have low HGT rates and little discordance beyond ILS (increasing from 30% with ILS-only to 34%). However, the last two conditions have substantially higher HGT rates, with 53.4% and 68.4% total discordance, making for more challenging input (Table S5).

The log error for all methods has almost no change across the four easy conditions, and the MAE fluctuates within the bounds of standard error (Fig. S14A). Comparing the last three conditions, both log and MAE for all methods generally increase for higher HGT rates. Distance-based methods are generally the least accurate across different conditions, except for the highest HGT rate, where concatenation has a higher MAE. CASTLES-Pro is the most accurate for both metrics across all conditions, except for the highest HGT rate, where it has a tie with CASTLES in terms of log error. While TCMM alone is inaccurate, following CASTLES-Pro by regularized TCMM, as detailed by Arasti et al. (2024), further improves its accuracy and obtains the best results overall. We note that TCMM internally has an outlier removal step, which may be effective for dealing with HGT, but does not have any theoretical justification for ILS. The gap between CASTLES-Pro (with or without TCMM) and concatenation widens as HGT increases in terms of MAE (i.e. focusing on long branches) but closes for mean log error (focusing on short branches).

In terms of bias, terminal and internal branches again show different patterns (Fig. S14B). CASTLES-Pro and CASTLES have an underestimation bias in all model conditions, especially for internal branches. This underestimation mostly disappears if CASTLES-Pro is followed by TCMM. Concatenation and, to a smaller degree, distance-based methods have a large overestimation bias for terminal branches that increases with HGT rates. The over-estimation of terminal branches by concatenation is, on average, 2.77 times larger than the underestimation for terminal or internal branches by CASTLES-Pro and 5.45 times larger than the underestimation bias of CASTLES-Pro+TCMM over all branches.

### Scalability

CASTLES-Pro also has a runtime advantage on the 100-taxon GDL datasets, and its advantage becomes more clear as the number of genes increases (Fig. S13). In particular, for 10,000 genes, CASTLES-Pro takes on average less than 1 minute to estimate branch lengths on a fixed tree topology, while CA-DISCO takes about 124 minutes (Table 2). The gap between CASTLES-Pro and CA-DISCO widens as gene family trees become larger (see Table S4 for the average number of leaves); with the highest duplication rate ($10^{-9}$) and no loss, CASTLES-Pro finishes in 4 minutes on average, while CA-DISCO takes more than 25 hours on average (Fig. S13). In terms of memory usage, CASTLES-DISCO and CASTLES-Pro are almost identical and use much less memory than other methods (Table 2 and Fig. S13). Finally, in the model condition with 1,000-taxon trees and 1,000 genes, CA-DISCO and distance-based methods fail due to the memory limit (128GB of RAM), while CASTLES-Pro and CASTLES-DISCO finish in 2 and 11 minutes on average, respectively, and use less than 4 GB of memory.

**Table 2.** Runtime and peak memory usage of different methods for 100-taxon GDL+ILS dataset for 10,000 genes with sequence length of 100 bp.

	time (minutes)	peak memory (GB)
CASTLES-Pro	0.96	4.09
CASTLES-DISCO	6.45	3.64
CA-DISCO(RAxML)	123.72	29.26
ERaBLE-DISCO	43.35	12.78
FastME(AVG)-DISCO	19.61	8.81

The duplication rate is $5\times 10^{-10}$ with equal loss rate. The results are averaged across 10 replicates. The runtime does not include gene tree estimation or species tree topology estimation time, as all methods draw branch lengths on a fixed tree topology. See also Fig. S13.

### Biological Datasets

We next applied CASTLES-Pro to nine biological datasets with different sources of gene tree discordance (Table 1, Table S6). We compare the branch lengths produced by CASTLES-Pro on ASTRAL or ASTRAL-Pro topologies to concatenation branch lengths drawn on either ASTRAL or concatenation topologies.

#### ILS

**Fig. 3.**
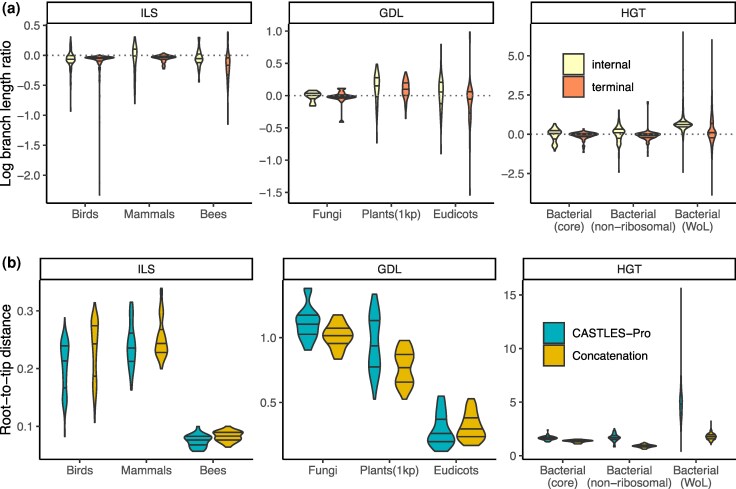
a) The branch lengths produced by CASTLES-Pro divided by branch lengths of concatenation on nine biological datasets with different sources of gene tree heterogeneity in log scale. b) Distribution of the root-to-tip distance for the CASTLES-Pro and concatenation trees on the nine biological datasets. See also Fig. S24.

As examples of datasets where biological discordance is likely dominated by ILS, we analyzed the birds dataset by Stiller et al. (2024), bees by Bossert et al. (2021), and mammals by Song et al. (2012), each of which had a published ASTRAL topology, which we used for both concatenation and CASTLES-Pro branch length estimation. On these datasets, we observe that CASTLES-Pro produces shorter lengths than concatenation, especially for terminal branches (Fig. 3). This pattern is more extreme for the birds and bees datasets (13.8% and 10.6% increase in average root-to-tip distance, respectively), which have a particularly high level of observed gene tree discordance. In addition, changes are more pronounced for terminal branches (Fig. 3) and especially short terminal branches (Figs. S15 and S16), as expected by theory and results of the simulations.

For terminal branches of the bird dataset, concatenation has a slightly shorter length for only one species and substantially longer lengths for others. The median (1st and 3rd quantiles) reduction from concatenation to CASTLES-Pro lengths is 0.00333 SU (0.00247, 0.00454); if we assume a mean substitution rate of 0.0026 per million years as estimated by Stiller et al. (2024) and 6.6 years per generation (median across species reported by Houde et al. 2020), the gap between concatenation and CASTLES-Pro corresponds to the expected 1 CU if $N_{e}=\frac{0.00333}{0.0026}\times \frac{10^{6}}{6.6}=194k$ (144k, 265k), which matches previous estimates using PSMC (Nadachowska-Brzyska et al. 2015).

Similarly, for bees, there are only three shorter terminal branches in concatenation compared to CASTLES-Pro, and one of them (*S. schubotzi*) becomes substantially longer. This increase is due to one gene tree with the clearly incorrect terminal length of 2.63 for *S. schubotzi*. Removing this single gene tree or using TreeShrink (Mai and Mirarab 2018) to filter abnormally long branches both reduce the length of this branch in the CASTLES-Pro output from 0.0454 to 0.0145 or 0.0157, which are below concatenation (Fig. S15). Using original gene trees (including outliers), we see the expected decrease in terminal lengths, with a median of 0.00255 (0.00159, 0.0047), which, divided by the spontaneous mutation rate of $3.5\times 10^{-9}$ per generation given by Wallberg et al. (2015), corresponds to $N_{e}=729k$ (452k, 1,350k) for 1 CU, which is in line with estimates based on PSMC (Lozier et al. 2023).

Finally, for mammals, four terminal branches become longer in CASTLES-Pro (including one with mislabeled taxa) while others become shorter. The elongated four are due to outliers, as using TreeShrink shortens all four branches, with two becoming shorter than concatenation and the other two remaining only 0.5% and 1.4% longer than concatenation. Even without TreeShrink, terminal branches shrink in CASTLES-Pro by a median of 0.0036 (0.0014, 0.0052), which assuming a per generation mutation rate of $2.5\times 10^{-8}$ (Pfeifer 2020) would correspond to 1 CU for $N_{e}=145k$ (57k, 206k).

#### GDL

For GDL, we analyzed two plant datasets (Wickett et al. 2014; Chanderbali et al. 2022) and a fungal dataset (Butler et al. 2009) that included multi-copy gene family trees. Since directly using concatenation on multi-copy gene sequences was not possible, we compared the concatenation topology on single-copy genes from original studies to ASTRAL-Pro topology furnished with CASTLES-Pro branch lengths run on multi-copy genes (the two trees differed by 8–9% RF). In these cases, we focus on branches that are shared between the two trees. For fungi, we inferred a tree using ASTRAL-Pro2 (Zhang and Mirarab 2022) using all multi-copy gene trees. On this dataset, the original study ran concatenation on 30,000 sites sampled from 706 single-copy orthologs, to which we compare. For 1KP, single-copy concatenation and multi-copy ASTRAL-Pro trees have 80 taxa in common; we induce all trees down to these taxa.

Patterns on GDL datasets differ from ILS-dominated datasets (Fig. 3), perhaps because here concatenation is run on single-copy genes while CASTLES-Pro is run on the full set of multi-copy gene trees. On the 1KP plant dataset, CASTLES-Pro run on 9,610 multi-copy gene trees has longer terminal and internal branch lengths than concatenation run on 424 single-copy genes (Fig. 3 and Fig. S19), leading to 24.2% higher mean root-to-tip distance. Similarly, on the fungal dataset, CASTLES-Pro based on 7,180 multi-copy gene family trees produces longer branches and 10.1% higher average root-to-tip distance compared to concatenation on 706 single-copy genes (Fig. 3 and Fig. S20). On the small and less diverse 40-taxon eudicots dataset, CASTLES-Pro based on 2,573 multi-copy gene trees results in shorter terminal branches but longer internal branch lengths than concatenation based on 345 single-copy genes (Fig. 3 and Fig. S18), with 7.7% decrease in average root-to-tip distance. Thus, patterns were different between these two plant datasets, and patterns were unlike ILS datasets; we return to this point in the discussions.

#### Microbial Data and AB Branch

A long-standing hypothesis has been that bacteria and archaea domains are separated by a long branch (Gogarten et al. 1989; Iwabe et al. 1989; Cox et al. 2008). In contrast, Zhu et al. (2019) (which included some of us) estimated a far shorter length for the AB branch than what was previously reported using a concatenation of 381 marker genes for the branch length estimation step. Moody et al. (2022) further studied this and other microbial datasets and suggested (among other criticisms) that concatenation can severely underestimate branch lengths on datasets with high levels of HGT, resulting in underestimation of the AB branch length. They report estimates of AB branch as long as 3.3 based on the core gene set of Williams et al. (2020) and 2.52 using the 27 most vertically evolving genes selected from a set of manually curated marker genes, including both ribosomal and non-ribosomal proteins. We reexamine three bacterial datasets (Petitjean et al. 2015; Zhu et al. 2019; Williams et al. 2020) that Moody et al. (2022) also studied. For small bacterial datasets, we inferred a species tree using ASTRAL-III (Zhang et al. 2018). For the WoL bacterial dataset, the original study had a species tree, but for branch lengths, Zhu et al. (2019) randomly selected 100 sites from sites with less than 50% gaps for each of the 381 marker genes (due to the high memory demand of concatenation).

**Fig. 4.**
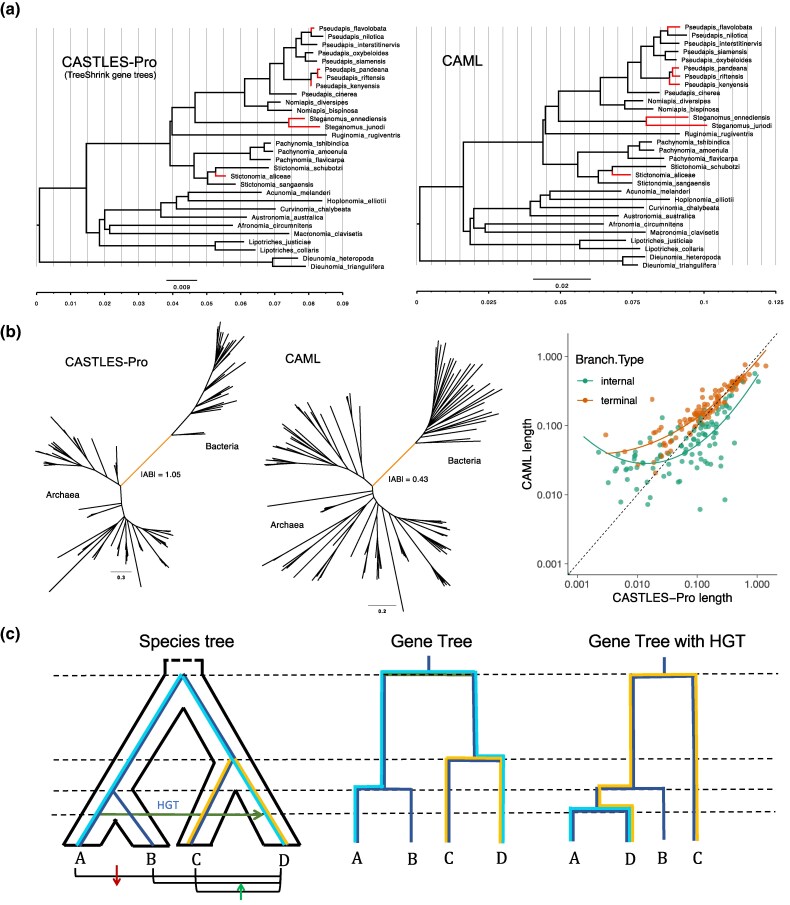
a) Comparison between the branch lengths of CASTLES-Pro (with TreeShrink gene trees) and CAML on the 32-taxon 853-gene bees dataset of Bossert et al. (2021) after removing the outgroup taxa *Lasioglossum albipes* and *Dufourea novaeanglia*. We used the ASTRAL topology from the original study and used concatenation and CASTLES-Pro to draw branch lengths on this topology. The branch lengths that are at least 2x shorter in the CASTLES-Pro tree compared to the concatenation tree are highlighted in red. b) Comparison between the branch lengths produced by CASTLES-Pro and concatenation on the 108-taxon bacterial dataset with 38 non-ribosomal genes. The branch highlighted in orange separates Archaea from Bacteria (AB branch). c) HGT, if ignored (as in concatenation), can make branches longer or shorter; the HGT event shown by the dark green arrow creates the gene tree shown on the right, which has a shorter distance from A and B to D, but higher distances from D to C. This reduces branch length for some branches in the species tree (blue) and increases it for others (yellow). See also Figs. S15–S23.

On all three microbial datasets, we observe a general increase in the length of the internal branches, particularly longer branches, and a slight decrease in the length of terminal branches (particularly short ones) for CASTLES-Pro compared to concatenation (Fig. 3 and Fig. S23). Since internal branches increase more than terminal branches decrease, we observe a substantial increase in the average root-to-tip distance on all three datasets (Figs. 3, 4 and Figs. S21 to S23). For the WoL dataset, branches change dramatically between the methods; however, note that Zhu et al. (2019) limited itself to 100 sites per gene due to scalability limitations of concatenation, while CASTLES-Pro uses gene trees estimated from full-length sequence alignments. The concatenation tree has a long tail of branches with 1e-6 or 2e-6 length, corresponding to no-event branches among 100×381 sites chosen, but no such tail exists for CASTLES-Pro (Fig. S23) since it uses all sites.

On all three datasets, CASTLES-Pro substantially increased the AB length compared to concatenation (Table 3) and made them closer to those estimated by Moody et al. (2022). The increases are dramatic (17×) on the WoL dataset, which contains highly discordant genes, substantial (2.5×) on the less discordant non-ribosomal genes, and relatively small (1.3×) on the presumably HGT-free core genes. On the two small, less discordant datasets, there are orders of magnitude more matching quartets than non-matching quartets, signifying a lack of discordance, and in both cases, the length of matching quartets is much longer than non-matching ones (Table 3). In contrast, on WoL, the number of matching and non-matching quartets are both very large, and matching quartets are only 1.7x more than non-matching ones. Nevertheless, the length of the AB branch in all three cases remains close to the average AB length in the matching quartets. Overall, CASTLES-Pro produces longer internal branches and longer AB lengths for all three bacterial datasets compared to concatenation, a trend that agrees with the observations of Moody et al. (2022), who suggest that concatenation can underestimate branch length in the face of high HGT.

**Table 3.** AB branch length on the three bacterial datasets estimated by CASTLES-Pro, TCMM, and concatenation.

	${\bar{L}}_{I}$	${\bar{L}}_{I}^{\prime }$	$C_{M}$	$C_{NM}$	*d*	CASTLES-Pro	TCMM	CAML
Core	1.92	0.01	922,226	32	9.83	1.79	1.94	1.43
Non-ribosomal	1.06	0.08	1,270,289	11,624	4.30	1.05	0.87	0.43
WoL	2.74	0.74	750,795,570,342	444,568,398,602	0.58	2.68	1.89	0.16

${\bar{L}}_{I},{\bar{L}}_{I}^{\prime }$ refer to the average AB branch length in matching and non-matching gene trees, respectively, and $C_{M},C_{NM}$ refer to the number of matching and non-matching quartets around the AB branch. *d* refers to the length of the AB branch in coalescent units.

## Discussion

We introduced CASTLES-Pro, a summary method that can furnish a given species tree with substitution-unit branch lengths accounting for GDL and ILS based on a given set of potentially multi-copy gene trees. Our simulations with ILS alone or ILS+GDL showed that CASTLES-Pro is more accurate than other methods in most conditions and is also more scalable, easily running on datasets with tens of thousands of species or genes (Table S7) in less than an hour. In the face of HGT, which CASTLES-Pro does not directly model, it was still more accurate than concatenation but had room for improvement. In particular, using CASTLES-Pro with automated outlier removal methods reduced its bias for high HGT cases, a topic explored by Arasti et al. (2024). Paired with summary methods that infer the topology, this advance makes the two-step approach to species tree inference more practical than before. The ASTER package (Zhang et al. 2025) outputs trees with SU branch lengths when used with ASTRAL-IV (for single-copy genes) or ASTRAL-Pro-2 (for multi-copy genes) using the CASTLES-Pro algorithm. These trees can readily be used as input to downstream analyses such as dating.

The negative impact of concatenation on branch lengths depended on the cause of discordance, as biological analyses clearly show (Fig. 3). For ILS, as expected, terminal branch lengths, especially shorter ones, are over-estimated using concatenation, but internal branches have far less bias overall. This imbalance between the error for terminal and internal branches can lead to unexplainable patterns in downstream analyses, such as diversification rates (Arbogast et al. 2002; Burbrink and Pyron 2011; Mendes and Hahn 2016; Tabatabaee et al. 2025). Note that for internal branches, depending on the coalescent length of surrounding branches, we may still have overestimation or underestimation using concatenation, but looking across all internal branches, those effects diminish.

In contrast to ILS, on GDL datasets where CASTLES-Pro was given all loci and sites per locus and concatenation was based on fewer genes and sites, CASTLES-Pro generally produced longer branches. One explanation for the increase in branch lengths in GDL datasets is ascertainment bias. The small portion of loci that happen to be single-copy tend to be the most conserved ones, giving a biased picture of the substitution rates. By allowing the use of all multi-copy gene trees, CASTLES-Pro reveals higher genome-wide substitution rates compared to single-copy genes. An alternative explanation is that single-copy gene trees are easier to align due to being smaller (and more conserved), and the higher branch lengths from CASTLES-Pro could be a result of over-alignment in multi-copy genes or perhaps saturation. The real GDL data likely suffer from a mixture of both, especially given that angiosperm data (which span a far shorter evolutionary time) did not experience branch length increase.

For HGT, our simulations showed an over-estimation bias for concatenation, whereas, on real microbial data, we seemed to observe the opposite. For the AB branch (but also others), concatenation underestimates lengths compared to CASTLES-Pro and compared to using HGT-free genes. The difference is likely due to the type of HGT events. Any single HGT event both increases and decreases divergence for some pairs of taxa compared to the species divergence, leading to both under-estimation and over-estimation bias for different branches (see Fig. 4c). Our simulations using Simphy augment ILS with *random* HGT events, each of which can create bias in either direction for individual branches. When many such events accumulate, they can cancel each other out, leading to a less clear HGT signature and leaving us with impacts of ILS. Real biological data often experience highways of HGT when large numbers of genes are transferred between two points of the tree. Such highways are expected to have happened around the AB branch (Beiko et al. 2005; Puigbò et al. 2009). One expects HGT highways between two branches to create a strong under-estimation bias for branches connecting the donor and recipient; a large portion of the concatenated alignment will have reduced divergence between pairs of species, one from the donor and one from the recipient (see Fig. 4c). Our results are consistent with the claim by Moody et al. (2022) that the AB length seems to be underestimated by concatenation for this reason.

The underestimation around the AB branch seemed to be fixed in CASTLES-Pro, but the reason needs explanation since this phenomenon is separate from the coalescent dynamics CASTLES-Pro models. When a branch has a long estimated CU length, the relatively few gene tree quartets that disagree with the species tree can have abnormally long lengths, even exceeding the matching ones on average. This is exactly the situation for the AB branch (Table 3). In such cases, CASTLES-Pro resorts to using the mean length of matching gene tree quartets for the species tree and thus effectively ignores coalescent effects, which is defensible for a branch with a large CU length. In doing so, it eliminates gene tree quartets that disagree with the species tree, which are presumably due to HGT for the AB branch. This feature (and not expectations under coalescence) leads CASTLES-Pro to output a large distance for the AB branch in contrast to concatenation.

Our method paves the way for a new four-stage phylogenomics pipeline that uses scalable methods in each step: Estimate gene trees independently, estimate the species tree topology by summarizing the gene tree topologies, estimate the branch lengths using CASTLES-Pro (optionally followed by TCMM for high HGT), and date the tree using scalable methods such as TreePL (Smith and O’Meara 2012) or MD-CAT (Mai et al. 2024). With this pipeline, we can easily handle datasets with thousands of species and thousands of genes.

## Materials and Methods

### CASTLES-Pro Algorithm

We first review an extension of the MSC model that allows for varying substitution rates across the branches of the species tree and the CASTLES algorithm, on which CASTLES-Pro is based. We then describe how it is extended to handle multi-copy gene family trees and end by explaining the methods used to enhance CASTLES for both single-copy and multi-copy gene trees.

### MSC+Substitution Model

We consider a model that is parametrized by a species tree topology T, and for each branch i∈E(T), two parameters are defined: The CU length $T_{i}$ and per-branch substitution rate $\mu_{i}$ in the unit of substitutions per sequence site per CU. Thus, $\mu_{i}$ increases not only with per generation substitution rate $\nu_{i}$  *but also* with the effective population size $N_{e}$, so that $\mu_{i}=\nu_{i}N_{e}$ (or $\mu_{i}=2\nu_{i}N_{e}$ for diploids). This is a standard model used in simulations. For example, in Simphy simulations, $\nu_{i}$ corresponds to the tree-wide substitution rate per generation (-su) times Species-specific branch rate heterogeneity modifiers (-hs parameter), and $N_{i}$ is the effective population size parameter (-sp). Species tree branch lengths are multiplied by corresponding substitution rates to produce the species tree SU lengths $t_{i}=T_{i}\mu_{i}$ (Simphy has both $T_{i}$ and $\mu_{i}$ but did not output them separately, necessitating our modification to do so). We use $t=\langle t_{a},t_{b},\ldots \rangle$ and $\mu =\langle \mu_{a},\mu_{b},\ldots \rangle$ as shorthand for all SU lengths and mutation rates, respectively. The assumed generative model is as follows: Gene trees with CU branch lengths are generated from the species tree under the MSC model or an MSC+GDL model, such as DLCoal (Rasmussen and Kellis 2012). Then, each gene tree branch length is scaled by the weighted average of mutation rates corresponding to all branches in the species tree that it traverses (weights proportional to the time spent in each species tree branch). Gene trees can be each scaled with a locus-specific rate. Simphy simulations follow this process in the ILS-only simulations, and similar processes for ILS+GDL and ILS+HGT. In addition, Simphy also multiplies each branch of each gene tree independently by a (third) Gene-by-lineage-specific rate heterogeneity modifier (-hg), an additional source of variation that is present in our simulations but not the described model.

#### CASTLES

The input to CASTLES is a species tree topology and a set of *k* unrooted single-copy gene trees with branch lengths in units of expected number of substitutions per site. Its output is the species tree with SU lengths on all branches.

CASTLES is based on expected values of gene tree branch lengths under the MSC. Treating species tree SU branch lengths t and mutation rates μ as unknown parameters, we can analytically calculate the expected length of the gene tree branches. For a simple cherry tree (a,b):*T* with mutation rate $\mu_{a}$, $\mu_{b}$ for terminal branches and $\mu_{r}$ for the parent branch, this expected length is $\mu_{a}T+\mu_{r}=t_{a}+\mu_{r}$ and $\mu_{b}T+\mu_{r}=t_{b}+\mu_{r}$ for terminal branches of *a* and *b*, resp. Note that each terminal length has an extra $\mu_{r}$ term. This gap between speciation and genic divergence (1 CU in expectation; i.e. $N_{e}$ generations) is not modeled by concatenation or methods that do not account for coalescence and can impact downstream analyses such as divergence time estimation (Edwards and Beerli 2000; Arbogast et al. 2002; Ho et al. 2005; Mendes and Hahn 2016).

**Fig. 5.**
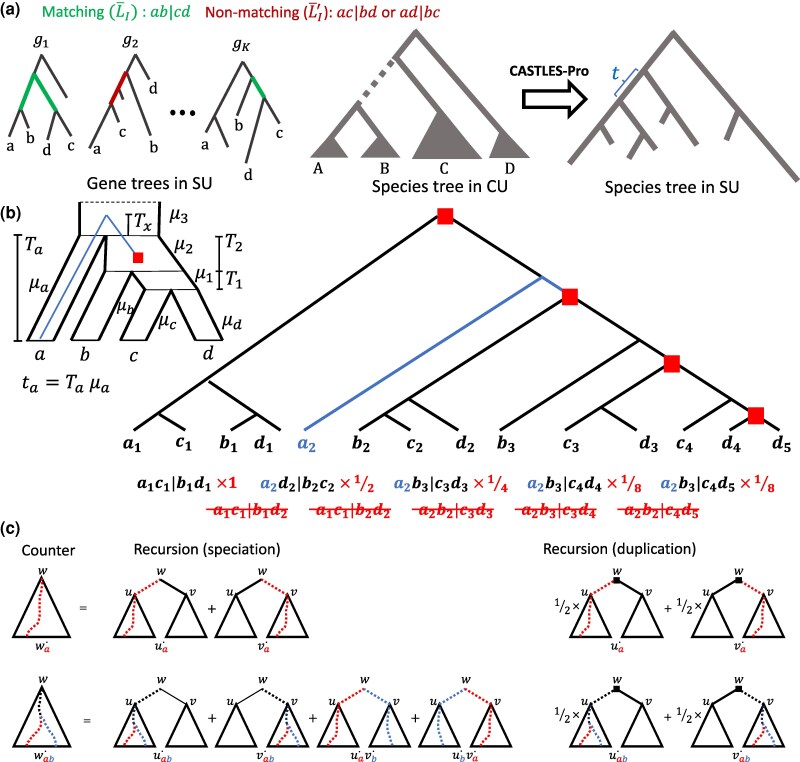
a) Illustration of the CASTLES-Pro algorithm. For every quartet a∈A,b∈B,c∈C,d∈D around a focal branch of the species tree (dotted), an unrooted quartet in a gene tree either matches it (ab|cd) or does not (ac|bd). The internal edge of the quartet maps to a path in the gene tree; the average of the path length in gene trees that do (${\bar{L}}_{I}$) and do not ($\bar{L_{I}^{\prime }}$) match the species tree are used to compute the branch length of the species tree in the SU unit (*t*). b) Illustration of an unbalanced species tree and a gene family tree undergoing consecutive duplication events noted in red boxes. Each species tree branch has a substitution rate $\mu_{i}$ (substitutions per site per CU). A gene tree branch takes the average rate of species tree branches it passes through, weighted by time spent in each branch; e.g. the rate of $a_{2}$ terminal branch is $\left(\mu_{a}T_{a}+\mu_{3}T_{x})/(T_{a}+T_{x}\right)$. We show all quartet trees involving only orthologous genes alongside their respective weights, followed by examples of quartet trees that include paralogous genes (not counted). The weight of a quartet is $2^{-d}$, where *d* is the number of duplication events the quartet passes through. The sum of all quartets that include a branch (e.g. $a_{2}$), is at most 1 but can be lower (e.g. 1/2 for $b_{3}$). c) Examples of counters for computing the weighted count of gene tree quartets using dynamic programming (see Supplementary Fig. S1 for all cases). For the species tree quadripartition AB|CD, we compute $L_{I}$ and $L_{I}^{\prime }$ in a bottom-up traversal. At each node *w*, several counters are updated; the two simplest counters are shown: $w_{a}^{\cdot }$ (weighted number of leaves corresponding to a∈A below *w*) and $w_{ab}^{\cdot }$ (weighted number of pairs (a,b)∈A×B with a speciation node as LCA at or below *w*).

We can extend this idea to quartet trees using more advanced calculations and distinguishing gene trees that match or do not match the species tree. These equations would be of the form E(L)=f(t,μ) where *L* is a random variable representing the length of a terminal ($L_{T}$) or internal ($L_{I}$) branch of a quartet gene tree matching the topology of the species tree or conflicting with it ($L_{I}^{\prime }$ and $L_{T}^{\prime }$). We derived those equations, reproduced in Tables S1 to S3. To estimate species tree SU lengths given a set of gene trees, we first compute the average branch lengths for gene trees that match or conflict with the topology of the species tree (${\bar{L}}_{I}$, ${\bar{L}}_{T}$, ${\bar{L}}_{I}^{\prime }$, ${\bar{L}}_{T}^{\prime }$); see Fig. 5a. Equating the theoretical expected values (with unknown parameters) with observed values, we get a set of equations that can be analytically solved. CASTLES employs several simplifying assumptions to reduce the number of parameters further and simplify the equations (Table S2). For example, to estimate an internal branch with length $t_{1}=T_{1}\mu_{1}$ (Fig. 5b), we obtain


(1) $$\bar{\delta }\;:\;=\frac{{\bar{L}}_{I}}{{\bar{L}}_{I}^{\prime }}-1=\frac{3(T_{1}+e^{-T_{1}}-1)}{3-2e^{-T_{1}}}$$

which we solve for $T_{1}$ to obtain


(2) $$\hat{T_{1}}=g(\bar{\delta })\;:\;=\bar{\delta }+W\left(-\frac{1}{3}e^{-\bar{\delta }-1}(2\bar{\delta }+3)\right)+1$$

where W(.) is the Lambert *W* function. We then estimate substitution rate $\mu_{1}$ using a second equation with further simplifying assumptions (see Supplementary Section B) to obtain $\hat{\mu_{1}}={\bar{L}}_{I}^{\prime }$ and thus:


(3) $$\hat{t_{1}}=g(\bar{\delta }){\bar{L}}_{I}^{\prime }$$

CASTLES extends these calculations to n>4 species by computing averages over all quartets around each species tree branch, a task that can be done in $O(n^{2})$ using a dynamic programming algorithm.

#### Handling Duplication and Loss

Gene duplications create quartet trees with paralogous gene copies. CASTLES-Pro addresses this issue by striving to exclusively use quartets devoid of paralogous genes. Following ASTRAL-Pro2, we first tag each internal node of each input gene tree as either a duplication or a speciation event, using the same parsimony method used by ASTRAL-Pro; we assume these *tags* are accurate (in practice, they are obtained using parsimony and may have errors). CASTLES-Pro operates similarly to CASTLES, with two major changes. The main change is that a quartet contributes to empirical mean branch length (${\bar{L}}_{I}$, ${\bar{L}}_{T}$, ${\bar{L}}_{I}^{\prime }$, ${\bar{L}}_{T}^{\prime }$) only if the least common ancestors (LCAs) of all pairs of its leaves are speciation nodes (these are called orthologous quartets). If all the tags are correct, orthologous quartets will follow the MSC expectations (Zhang et al. 2020) and, thus, CASTLES assumptions.

The second change relates to the potentially uneven rates of duplication across gene families, which can lead to certain branches being overrepresented in the final means. Figure 5b demonstrates an example; four out of five orthologous quartets share the $a_{2}$ branch indicated in blue; however, the duplication events are on a separate branch and result in using $a_{2}$ four times in computing mean branch lengths of *a* (e.g. ${\bar{L}}_{I}$) for no apparent reason. Counting all orthologous quartets without weights can increase the impact of individual branches and, thus, the variance in estimated means. We use a weighting scheme to mitigate the impact of this overrepresentation. The weights are simply set to $2^{-d}$ where *d* is the number of duplication nodes falling on the subtree spanned by the quartet tree. With this scheme, it is easy to show that the total weight of quartets that include a branch will not exceed 1 in any gene family tree.

Beyond eliminating apparent paralogs and weighting ortholog quartets, the main challenge is computing mean branch lengths efficiently instead of the trivial $O(n^{4})$ algorithm that lists all quartets. We designed a dynamic programming algorithm that achieves this goal in $O(n^{2})$ time (see Supplementary Section A and Algorithm S1). Compared to CASTLES, we needed to refine the set of our counters updated in the dynamic programming (demonstrated in Supplementary Figs. S1 and S2.) Crucially, at duplication nodes, the recursive formulas change to ignore non-orthologous quartets and implement the weights (see Fig. 5b). After calculating the means, CASTLES-Pro assigns lengths to all branches of the species tree in an O(n) pre-order traversal of the tree, and therefore the total runtime of the CASTLES-Pro algorithm is $O(n^{2})$.

#### Better Approximations, Assumptions, and Handling of Short Branches

Even for single-copy gene trees, CASTLES-Pro improves on CASTLES in three ways and, as we will see, dominates it in terms of accuracy (thus, CASTLES-Pro replaces CASTLES). One change is related to a simplifying assumption: CASTLES-Pro uses a slightly different approach for handling dependencies on the parent branch when calculating the length of a terminal branch of a cherry. Due to space limits, this change is explained in supplementary material (Sec. B). We elaborate here on the other two changes.

CASTLES employed a weak approximation that we eliminate in CASTLES-Pro. Instead of directly using the Lambert function *W* in Equation (2), CASTLES used a Taylor approximation to get $g(\bar{\delta })\approx \frac{1}{2}\bar{\delta }+\frac{1}{6}\sqrt{3\bar{\delta }(3\bar{\delta }+4)}$. However, this approximation underestimates the true value as we move away from its focal point (0), causing a systematic underestimation bias (Fig. S4). Oddly, our simulation studies show that the Taylor approximation works better in practice on estimated gene trees despite this bias. Attributing this odd observation to difficulties with numerical precision, in CASTLES, we opted to use the Taylor expansion. However, we have now discovered a different explanation for this pattern.

Our simulations show that when using true gene trees, the Lambert *W* function is superior, whereas Taylor gradually becomes better as gene tree estimation error (GTEE) increases (Fig. S5). Taylor’s better performance is due to the interplay between two opposing sources of bias. As GTEE increases, we tend to overestimate $\bar{\delta }$, a pattern that can be explained. With a low phylogenetic signal and hence high GTEE, many of the short internal branches, which are more prevalent among gene trees not matching the species tree, become zero-event (i.e. record no substitutions). The inferred length of these supershort branches is often driven by a pseudocount used in the maximum likelihood tools (e.g. 1e-6), which is often an underestimation of the true value. Since ${\bar{L}}^{\prime }<\bar{L}$ to begin with, these underestimations happen more for ${\bar{L}}^{\prime }$ than $\bar{L}$, and thus, $\bar{\delta }=\frac{{\bar{L}}_{I}-{\bar{L}}_{I}^{\prime }}{{\bar{L}}_{I}^{\prime }}$ tends to get overestimated, which in turn offsets the underestimation bias of the Taylor approximation. This lucky cancellation is not enjoyed if we use the exact Lambert W function. As the signal increases, or with true gene trees, there is no overestimation to offset Taylor’s bias, leading to the exact Lambert equation working better.

In CASTLES-Pro, we directly address the overestimation of $\bar{\delta }$ under high GTEE conditions and switch to using the exact Lambert W function in Equation (2). If the length of an alignment is *s*, a branch of length 1/s would expect to see one substitution. Thus, branches substantially below 1/s are often zero-event and underestimated by a pseudocount. To address this, we simply add a pseudocount of 1/s to both ${\bar{L}}_{I}$ and ${\bar{L}}_{I}^{\prime }$, obtaining an adjusted value for *δ* given by $\delta_{C}=\frac{{\bar{L}}_{I}-{\bar{L}}_{I}^{\prime }}{{\bar{L}}_{I}^{\prime }+1/s}$. This is, in principle, similar to adding a pseudocount to binomial parameter estimation, which is equivalent to a posterior estimate under a Dirichlet prior. As sequence length increases and GTEE decreases, so does the pseudocount (in the limit, s=∞ for true gene trees, giving a zero pseudocount). The value of *s* can be adjusted by the user (default: 1,000).

Another difficulty arises when $\bar{\delta }$ is negative, indicating that the average length in non-matching gene trees is larger than that of matching gene trees. This is unexpected under MSC and will not happen with infinitely many error-free gene trees; in practice, however, it can happen for many reasons. CASTLES simply resorted to replacing $\bar{\delta }<0$ with a fixed pseudocount of $\delta_{p}\;:\;=10^{-3}$. For some causes of negative $\bar{\delta }$, this is not a good approach. When a branch is very long with a low level of ILS, there are very few non-matching gene trees. Furthermore, these non-matching gene trees can differ from the species tree due to reasons other than ILS, such as paralogy, horizontal transfer, incorrect homology, etc. Thus, the (few) non-matching gene trees can have average lengths that are larger than the average length of matching gene trees, leading to a negative $\bar{\delta }$. In such cases, simply using the mean of matching gene trees (${\bar{L}}_{I}$) is a better approximation. In contrast, the CASTLES approach of using a small pseudocount $\delta_{p}$ in the original equation $g(\delta_{p}){\bar{L}}_{I}^{\prime }$ makes sense for very short branches. CASTLES-Pro handles negative $\bar{\delta }$ using a formula that takes the level of ILS into account and transitions between these two approaches. For $\bar{\delta }<0$ we use:


(4) $$\hat{t_{1}}=\frac{\omega_{d}}{\omega_{d}+\frac{1}{\omega_{d}}}{\bar{L}}_{I}+\frac{\frac{1}{\omega_{d}}}{\omega_{d}+\frac{1}{\omega_{d}}}g(\delta_{p}){\bar{L}}_{I}^{\prime }$$

where $\omega_{d}={log}_{10}(k)d$ is the weight of the two formulas; *d* is the quartet-based CU length of the branch (see Sayyari and Mirarab 2016) and *k* is the number of gene trees. As gene tree discordance decreases, *d* and $\omega_{d}$ increase and in the limit, $\lim_{d\to \infty }\hat{t_{1}}={\bar{L}}_{I}$ in Equation (4); this is justified because for long branches, the deep coalescence has a *relatively* small impact compared to the full length. For short branches, *d* decreases, and in the limit $\lim_{d\to 0}\hat{t_{1}}=g(\delta_{p}){\bar{L}}_{I}^{\prime }$, we resort to the original formula (3) used with the pseudocount. Thus, we transition from relying on average matching gene tree length (${\bar{L}}_{I}$) for branches with little discordance to our original estimate for high discordance; the rate of transitioning between the two equations is governed by the number of genes, with more genes leading to faster adoption of ${\bar{L}}_{I}$; this is because the discordance-based estimates of CU length (*d*) are more accurate with more genes.

### Experimental Study

We provide high-level descriptions below and include additional details in Supplementary Section C.

We studied three sets of simulated datasets with gene tree discordance due to ILS, ILS+GDL, and ILS+HGT (Table 1). All simulated datasets are generated using SimPhy (Mallo et al. 2016); however, we modified SimPhy to output model species trees with SU branch lengths using mutation rates already present in SimPhy simulations. Gene sequences were simulated under the GTR+Γ model, and gene trees were estimated from these alignments using FastTree-2 (Price et al. 2010). The ILS-only dataset was reused from Tabatabaee et al. (2023) and has gene alignments of length 200 bp–1,600 bp to control gene tree estimation error (GTEE). The level of ILS is heterogeneous across replicates, with mean equal to 46% according to average Robinson and Foulds (1981) (RF) distance between model species trees and true gene trees (AD for short). The GDL+ILS dataset was reused from Willson et al. (2022, 2023) and has two levels of ILS (low and high), six duplication rates ($10^{-13}$–$10^{-9}$), three sequence lengths, various numbers of species, and genes (Table 1). The loss rate relative to the duplication rate is set to 1, 0.5, or 0. For ILS+HGT, we recreated a dataset by Davidson et al. (2015) with 30% AD due to ILS and six levels of HGT rates, leading to up to 68% AD (Table S5). The average number of HGT events per gene for the six model conditions starts from 0 to 0.08, 0.2, 0.8, 8, and 20, corresponding to HGT rates $10^{-9}\times (0,2,5,20,200,\;\text{and}\;500)$.

The methods we compare to are all designed for single-copy gene trees. For datasets with GDL, we create a two-step pipeline where we first use the method DISCO (Willson et al. 2022) to decompose gene family trees into single-copy gene trees, which we then pass to branch length estimation methods. These two-step methods are referred to as CASTLES-DISCO, ERaBLE-DISCO, and FastME(AVG)-DISCO. To perform concatenation with multi-copy input, we use the CA-DISCO technique of Willson et al. (2022). Sequences for each gene family are broken up into single-copy loci, and these loci are concatenated into a super-alignment. We use RAxML on this alignment to optimize branch lengths on the fixed true species tree topology. Note that DISCO can produce trees with high levels of missing data, and ERaBLE and FastME(AVG) can fail on inputs with missing data. To enable these methods to run on DISCO output, we imputed the missing values in the distance matrix of each gene tree by the average patristic distances among gene trees that do include the pair of taxa associated with the missing value. Finally, while the species tree estimation method SpeciesRax (Morel et al. 2022) can produce branch lengths in substitution units, we did not include it in this study as it cannot estimate branch lengths on a fixed input topology.

## Supplementary Material

evaf200_Supplementary_Data

## Data Availability

CASTLES-Pro is implemented inside the software package ASTER, available at https://github.com/chaoszh  ang/ASTER. The datasets and scripts used in this study are available at https://github.com/ytabatabaee/CASTLES-Pro-paper.
