# Supplementary material for: Species Tree Branch Length Estimation despite Incomplete Lineage Sorting, Duplication, and Loss
Source: Genome Biol Evol. 2025 Nov 26;17(11):evaf200. doi: 10.1093/gbe/evaf200 (PMC12648238; doi:10.1093/gbe/evaf200)
Supplement: evaf200_Supplementary_Data [file evaf200_supplementary_data.pdf]

Supplementary Materials for "Species tree branch length estimation despite incomplete lineage sorting, duplication, and loss"

Yasamin Tabatabaee[1], Chao Zhang[2], Shayesteh Arasti[3], Siavash Mirarab[4]

[1] Department of Computer Science, University of Illinois at Urbana-Champaign, Urbana, IL, USA
[2] GLOBE Institute, University of Copenhagen, Copenhagen, Denmark
[3] Department of Computer Science and Engineering, University of California San Diego, CA, USA
[4] Department of Electrical and Computer Engineering, University of California San Diego, San Diego, CA, USA

Contents

S1 Details of the Recursive Algorithm **3**

S2 CASTLES-Pro's Equations **7**

S3 Details of the Experimental Study **10**
S3.1 Simulated Datasets 10
S3.2 Biological Datasets 12
S3.3 Methods and Software Commands 14

S4 Additional Results (Figures and Tables) **16**

List of Figures

S1 Counters for computing the weighted count of gene tree quartets 5
S2 Counters for computing the weighted count of gene tree quartets (continued) 6
S3 Model quartet species tree and matching and non-matching gene trees inside it. 7
S4 Lambert W function vs its Taylor approximation for caluclating the internal branch . . . 16
S5 Comparing variants of CASTLES-Pro and CASTLES on S100 dataset. 17
S6 Comparing CASTLES-Pro with CASTLES-Pro +TCMM on S100 dataset. 18
S7 Mean absolute error, log error and bias on the GDL datasets for varying duplication rates. 19
S8 Mean absolute error, log error and bias on the GDL datasets for varying number of genes. 20
S9 Mean absolute error, log error and bias on the GDL datasets for varying number of genes. 21
S10 Mean absolute error, log error and bias on the GDL datasets for varying sequence lengths. 22
S11 Mean absolute error, log error, and bias on the GDL datasets for a varying number of species. 23
S12 Mean absolute error, log error and bias on the GDL datasets for varying dup/loss ratios. . 24
S13 Runtime and memory of branch length estimation methods on 100-taxon GDL datasets. . 25
S14 Mean absolute error, mean log error, and bias of methods on HGT datasets. 26
S15 Correlation between branch lengths of CASTLES-Pro and CAML on the bees datasets. . 27
S16 Correlation between branch lengths of CASTLES-Pro and CAML on the Neoavian dataset. 28
S17 Correlation between branch lengths of CASTLES-Pro and CAML on the mammals dataset. 29
S18 Correlation between branch lengths of CASTLES-Pro and CAML on the Eudicots dataset. 30
S19 Correlation between branch lengths of CASTLES-Pro and CAML on the plants dataset. . 31
S20 Correlation between branch lengths of CASTLES-Pro and MrBayes on the fungi dataset. 32
S21 Correlation between branch lengths of CASTLES-Pro and CAML on the bacterial dataset with core genes. 33
S22 Correlation between branch lengths of CASTLES-Pro and CAML on the bacterial dataset with non-ribosomal genes. 34

S23 Correlation between branch lengths of CASTLES-Pro and CAML on the WoL dataset. . . 35
S24 Comparison between the branch lengths of CASTLES-Pro and CAML on biological datasets. 36

List of Tables

S1 Summary of exact formulas for expected branch lengths in gene trees. 8
S2 Summary of simplified formulas for expected branch lengths in gene trees. 8
S3 Summary of formulas for estimating species tree branch lengths in SU. 9
S4 Empirical statistics of the simulated GDL datasets 11
S5 Empirical statistics of the simulated HGT datasets 11
S6 Empirical statistics of the biological datasets. ILS, GDL and HGT refer to the main source of gene tree discordance in each dataset. 13
S7 Runtime and peak memory usage of CASTLES-Pro on the biological datasets 37

S1 Details of the Recursive Algorithm

In this section, we will first provide an $O(n^2)$ algorithm for computing branch lengths for all internal and terminal branches. Notice, the complexity of this algorithm can be improved to $O(nH \log n)$, where H denotes the average height of gene family trees, using the dynamic programming algorithm in CASTLES. For conciseness, we use A, B, C, D to denote sets of taxa and use a, b, c, d to denote individual taxa. Let $\mathcal{G}$ be the set of gene trees.

To compute all branch lengths, it is sufficient to compute the following counters for a set of ordered leafset quadripartitions, in which each leafset quadripartition (A, B, C, D) – up to permutations – corresponds to an internal branch:

- $n(A, B; C, D)$: the number of quartet and gene tree combinations $(a, b, c, d, G) \in A \times B \times C \times D \times \mathcal{G}$ such that $G \upharpoonright \{a, b, c, d\}$ has topology $ab|cd$.
- $x(A, B; C, D)$: the total internal branch lengths of quartet trees in the form of $G \upharpoonright \{a, b, c, d\}$ with topology $ab|cd$, where $(a, b, c, d, G) \in A \times B \times C \times D \times \mathcal{G}$.
- $a(A; B; C, D)$: the total length of the terminal branches leading to A in quartet trees in the form of $G \upharpoonright \{a, b, c, d\}$ with topology $ab|cd$, where $(a, b, c, d, G) \in A \times B \times C \times D \times \mathcal{G}$.

All three counters for each quadripartition (A, B, C, D) can be computed in a single post-order traversal of the gene tree nodes in $O(n)$ using Algorithm S1. Therefore, computing counters for all $O(n)$ quadripartitions has time complexity $O(n^2)$. Notice that Algorithm S1 assumes that all input gene trees are fully resolved, consistent with the presumption of ASTRAL-Pro.

Algorithm S1 Recursive algorithm. The input is a set of gene trees $\mathcal{G}$ and an ordered quadripartition of the leafset (A, B, C, D), and the output are $n(A, B; C, D)$, $x(A, B; C, D)$, and $a(A; B; C, D)$. For each node u we keep a list of counters u^*_* described in Figs. S1 and S2.

procedure UpdateLeafCounters(u, A, B, C, D)
  Set all counters u^*_* to 0
  if u corresponds to a taxon in A **then**
    $u^{\cdot}_a \leftarrow 1$
    $u^a_a \leftarrow$ the parental branch length of u
  else if u corresponds to a taxon in B **then**
    $u^{\cdot}_b \leftarrow 1$
    $u^b_b \leftarrow$ the parental branch length of u
  else if u corresponds to a taxon in C **then**
    $u^{\cdot}_c \leftarrow 1$
    $u^c_c \leftarrow$ the parental branch length of u
  else if u corresponds to a taxon in D **then**
    $u^{\cdot}_d \leftarrow 1$
    $u^d_d \leftarrow$ the parental branch length of u
  end if
end procedure
procedure RecursiveAlgorithm($\mathcal{G}$, A, B, C, D)
  Set $n(A, B; C, D)$, $x(A, B; C, D)$, $a(A; B; C, D)$, $b(A; B; C, D)$, $c(A; B; C, D)$, $d(A; B; C, D)$ to 0
  for each gene $G \in \mathcal{G}$ **do**
    for $u \in$ post order traverse of nodes of G **do**
      if u is a leaf node **then**
        UpdateLeafCounters(u, A, B, C, D)
      else
        Update counter $u^{\cdot}_a$ using the recursive formula in Fig. S1 ▷ ditto for $u^{\cdot}_b, u^{\cdot}_c, u^{\cdot}_d$
        Update $u^{\cdot}_{ab}$ using Fig. S1 ▷ ditto for $u^{\cdot}_{cd}$
        Update $u^{\cdot}_{ab|c}$ using Fig. S1 ▷ ditto for $u^{\cdot}_{ab|d}, u^{\cdot}_{cd|a}, u^{\cdot}_{cd|b}$
        Update $u^{\cdot}_{ab|cd}$ using Fig. S1
        Update counter u^a_a using the recursive formula in Fig. S2 ▷ ditto for u^b_b, u^c_c, u^d_d
        Update counter u^a_{ab} using Fig. S2 ▷ ditto for $u^b_{ab}, u^c_{cd}, u^d_{cd}$
        Update counter u^x_{ab} using Fig. S2 ▷ ditto for u^x_{cd}
        Update counter $u^a_{ab|c}$ using Fig. S2 ▷ ditto for $u^b_{ab|c}, u^a_{ab|d}, u^b_{ab|d}, u^c_{cd|a}, u^d_{cd|a}, u^c_{cd|b}, u^d_{cd|b}$
        Update counter $u^x_{ab|c}$ using Fig. S2 ▷ ditto for $u^x_{ab|d}, u^x_{cd|a}, u^x_{cd|b}$
        Update counter $u^d_{ab|c}$ using Fig. S2 ▷ ditto for $u^c_{ab|d}, u^b_{cd|a}, u^a_{cd|b}$
        Update counter $u^a_{ab|cd}$ using Fig. S2 ▷ ditto for $u^b_{ab|cd}, u^c_{ab|cd}, u^d_{ab|cd}$
        Update counter $u^x_{ab|cd}$ using Fig. S2
      end if
    end for
    $u \leftarrow$ the root of G
    $n(A, B; C, D) \leftarrow n(A, B; C, D) + u^{\cdot}_{ab|cd}$
    $x(A, B; C, D) \leftarrow x(A, B; C, D) + u^x_{ab|cd}$
    $a(A; B; C, D) \leftarrow a(A; B; C, D) + u^a_{ab|cd}$ ▷ ditto for $b(A; B; C, D), c(A; B; C, D), d(A; B; C, D)$
  end for
end procedure

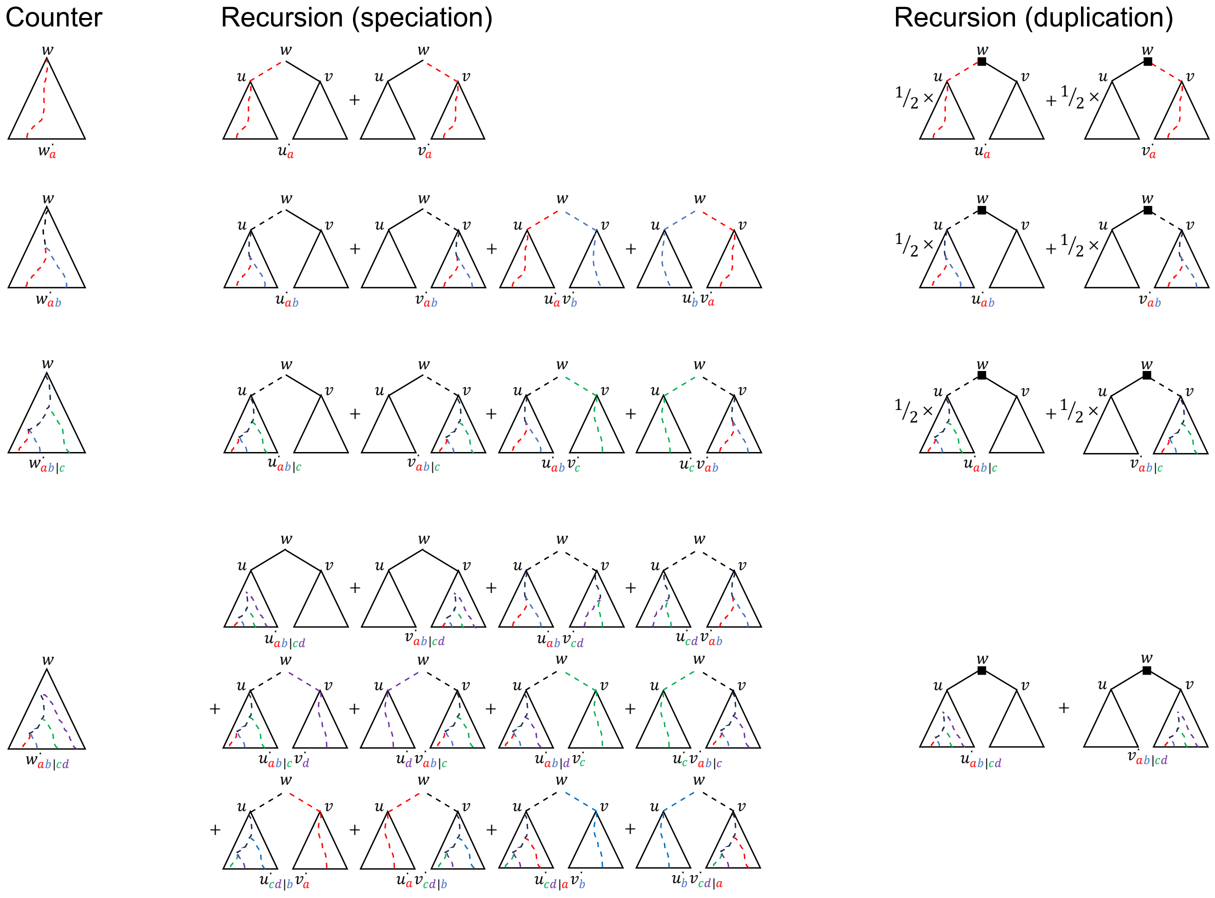

Figure S1: Additional counters for computing the weighted count of gene tree quartets aligning with the species quadripartition $ab|cd$. To the left presents a list of counters for each internal node w; in the middle illustrates how to recursively compute these counters from counters of the two children of w when w corresponds to a speciation event; to the right illustrates how to compute these counters when w corresponds to a duplication event. For example, $w^{\cdot}_{a} = u^{\cdot}_{a} + v^{\cdot}_{a}$ if w corresponds to a speciation event, and $w^{\cdot}_{a} = {}^{u^{\cdot}_{a}}/2 + {}^{v^{\cdot}_{a}}/2$ if w corresponds to a speciation event (u and v denote the children of w). The total weighted count of gene tree quartets aligning with the species quadripartition $ab|cd$ is calculated as $\sum_{w \in R} w^{\cdot}_{ab|cd}$, with R representing the set of root nodes across all gene trees.

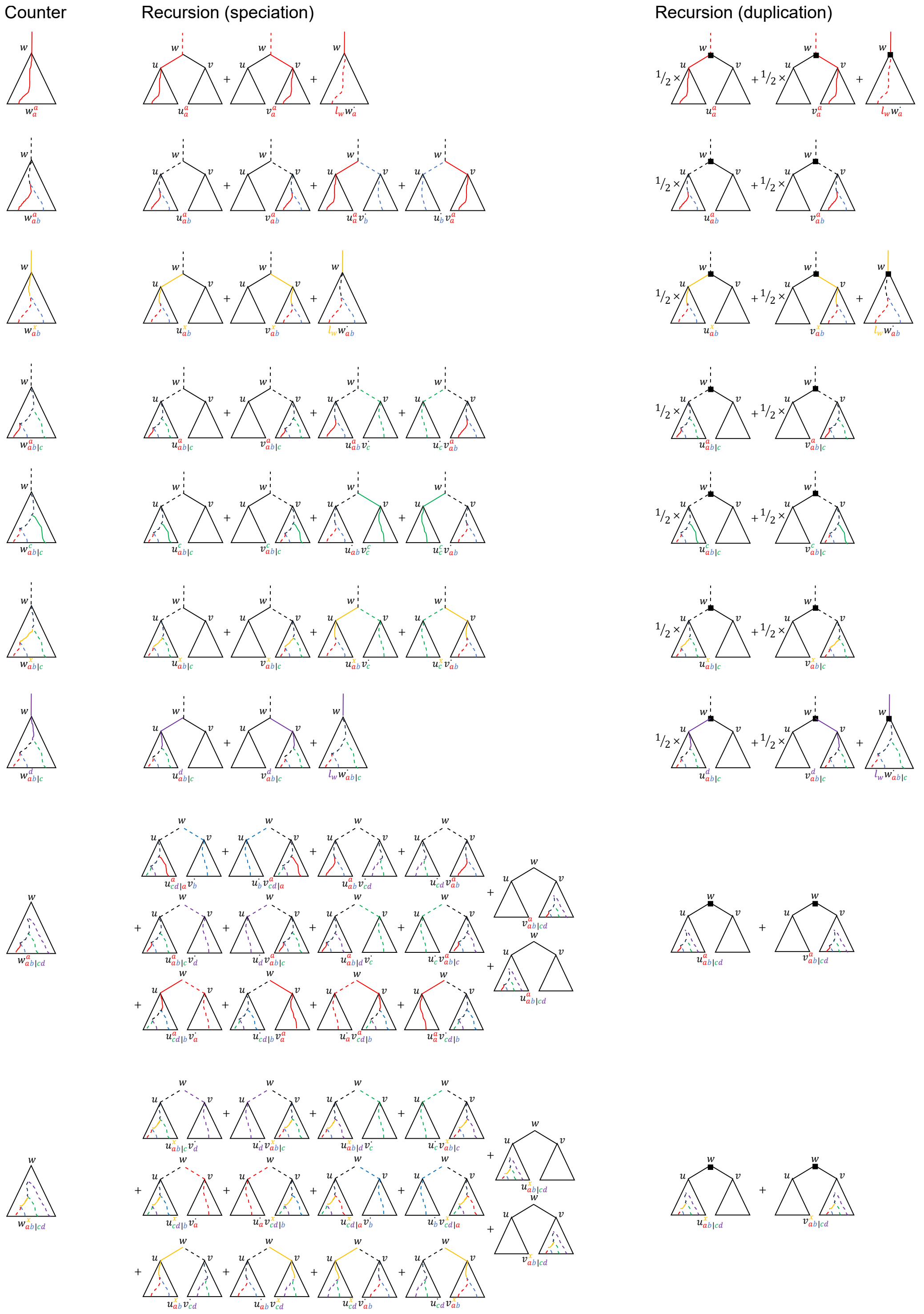

Figure S2: Counters for computing the weighted sums for internal and terminal branch lengths of gene tree quartets aligning with the species quadripartition $ab|cd$. The weighted mean internal branch lengths related to $ab|cd$ are $\sum_{w\in R} w^{x}_{ab|cd}/\sum_{w\in R} w^{\cdot}_{ab|cd}$ for the matching case and $\sum_{w\in R} w^{x}_{ac|bd}+w^{x}_{ad|bc}/\sum_{w\in R} w^{\cdot}_{ac|bd}+w^{\cdot}_{ad|bc}$ for the non-matching cases (similarly computed by permuting $a, b, c,$ and d). To compute the weighted mean for terminal branch lengths for the matching case, we use $\sum_{w\in R} w^{a}_{ab|cd}/\sum_{w\in R} w^{\cdot}_{ab|cd}$, $\sum_{w\in R} w^{b}_{ab|cd}/\sum_{w\in R} w^{\cdot}_{ab|cd}$, $\sum_{w\in R} w^{c}_{ab|cd}/\sum_{w\in R} w^{\cdot}_{ab|cd}$, and $\sum_{w\in R} w^{d}_{ab|cd}/\sum_{w\in R} w^{\cdot}_{ab|cd}$, respectively; to compute the weighted mean for terminal branch lengths for non-matching cases, we use $\sum_{w\in R} w^{a}_{ac|bd}+w^{a}_{ad|bc}/\sum_{w\in R} w^{\cdot}_{ac|bd}+w^{\cdot}_{ad|bc}$, $\sum_{w\in R} w^{b}_{ac|bd}+w^{b}_{ad|bc}/\sum_{w\in R} w^{\cdot}_{ac|bd}+w^{\cdot}_{ad|bc}$, $\sum_{w\in R} w^{c}_{ac|bd}+w^{c}_{ad|bc}/\sum_{w\in R} w^{\cdot}_{ac|bd}+w^{\cdot}_{ad|bc}$, and $\sum_{w\in R} w^{d}_{ac|bd}+w^{d}_{ad|bc}/\sum_{w\in R} w^{\cdot}_{ac|bd}+w^{\cdot}_{ad|bc}$, respectively.

S2 CASTLES-Pro's Equations

Tables S1-S3 summarize the exact and simplified formulas for expected branch lengths in matching and non-matching gene trees under the MSC, and final formulas for estimating branch lengths of an unbalanced or balanced quartet species tree that were used in CASTLES (Tabatabaee et al., 2023); in both figures, parameters are named according to Fig. S3. CASTLES-Pro uses much of the same formulas, but computes them in a different way that leads to improvements in accuracy. The changes for the internal branches (avoiding the Taylor approximation and ILS-aware weighting) are described in the main text, and here we describe the changes in calculating the terminal branches.

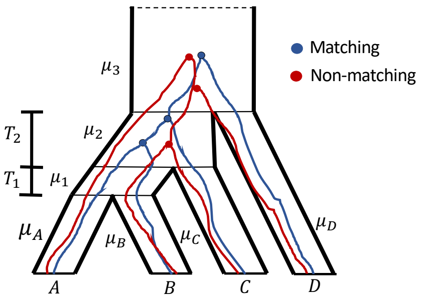

Figure S3: An unbalanced model species tree with four taxa with a matching and non-matching gene tree shown inside it. T_1 and T_2 denote the CU lengths, μ_is are the mutation rates, and the internal branch has an SU length of $t_1 = T_1\mu_1$ (the SU length for other branches are defined similarly). Taxa A and B are reffered to as cherry branches.

CASTLES uses Eq. (S1) to calculate the length of the cherry branch leading to taxa A (and similarly for B) in an unbalanced quartet species tree:

$$\hat{t_A} = \bar{L}'_A + \frac{\mu_1(e^{-T_1} - 1 + T_1) + \bar{\Delta}_A(1 - 2/3e^{-T_1})}{1 - 4/5e^{-T_1}} - T_1\mu_1\ . \tag{S1}$$

This equation depends on the mutation rate of the internal branch μ_1 and the CU branch length T_1. CASTLES uses the simplified formula for Δ_I in Table S2 to calculate μ_1 and substitutes it in Eq. (S1):

$$\hat{\mu_1} = \frac{\hat{\Delta}_I(3 - 2e^{-T_1})}{3(e^{-T_1} - 1 + T_1)}\ . \tag{S2}$$

Here, $\hat{T_1}$, the estimate of the CU length of the internal branch, is calculated using the approach from Sayyari and Mirarab (2016). However, as $T_1 \to 0$, the denominator of Eq. (S2) becomes very small, and therefore the formula becomes unstable as $\lim_{T_1 \to 0} e^{-T_1} = 1 - T_1$. In addition, the value of T_1 calculated using Sayyari and Mirarab (2016)'s algorithm degrades in accuracy as gene tree estimation error increases. While the formulas for the internal branches in CASTLES were calculated so as to not have a dependency on these CU lengths, the terminal branches still have this dependency.

To improve the estimation of μ_1 and reduce the dependency on the CU branch lengths estimated using Sayyari and Mirarab (2016)'s approach in Eq. (S1), CASTLES-Pro instead first calculates T_1 as a function of δ (see the main text) and μ_1 as $\bar{L}'_I$. It then directly uses these values to compute the terminal branch equations.

Table S1: Summary of formulas for expected branch lengths in matching and non-matching quartet gene trees. This table is reproduced from Table S1 in Tabatabaee et al. (2023).

Unbalanced

Parameter	Formula
L_I	$\frac{(e^{-3T_2}+3e^{-T_2}-6e^{T_1-T_2})(\mu_2-\mu_3)+6(1-e^{T_1}+T_1e^{T_1})\mu_1}{2(3e^{T_1}-2)} + \mu_2$
L'_I	$\mu_2 + \frac{1}{2}(\mu_2 - \mu_3)\left(e^{-3T_2} - 3e^{-T_2}\right)$
Δ_I	$\frac{3(e^{-T_2}-e^{-3T_2})(1-e^{-T_1})(\mu_2-\mu_3)+6\mu_1(e^{-T_1}-1+T_1)}{2(3-2e^{-T_1})}$
L_A	$\frac{6T_1\mu_1+3\mu_1-\mu_2+e^{-3T_2}(\mu_2-2\mu_3)}{6-9e^{T_1}} + \mu_1 + \mu_A T_A$
L'_A	$\frac{1}{12}(10\mu_2 - 9e^{-T_2}(\mu_2-\mu_3) - e^{-3T_2}(\mu_2+\mu_3)) + T_1\mu_1 + T_A\mu_A$
Δ_A	$\frac{\left(4\mu_2-6\mu_1-(3e^{-T_2}+e^{-3T_2})(\mu_2-\mu_3)+e^{T_1}\left(\frac{1}{2}(\mu_2+\mu_3)e^{-3T_2}+\frac{9}{2}e^{-T_2}(\mu_2-\mu_3)-6T_1\mu_1+6\mu_1-5\mu_2\right)\right)}{2(-2+3e^{T_1})}$
L_C	$-e^{-T_2}(\mu_2-\mu_3) + \mu_2 + \mu_C T_C + \frac{2\mu_2-\left(3e^{-T_2}-e^{-3T_2}\right)(\mu_2-\mu_3)-4\mu_3e^{-3T_2}}{6(3e^{T_1}-2)}$
L'_C	$\frac{1}{3}\mu_2(1+e^{-3T_2}) + \mu_C T_C$
Δ_C	$\frac{(2-e^{-T_1})\left(\left(e^{-3T_2}+2\right)\mu_2-3e^{-T_2}(\mu_2-\mu_3)\right)+\mu_3e^{-3T_2}(e^{-T_1}-4)}{2(3-2e^{-T_1})}$
L_D	$e^{-T_2}(\mu_2-\mu_3) - \mu_2 + 2\mu_3 + T_2\mu_2 + \mu_D T_D + \frac{-2\mu_2+\left(3e^{-T_2}-e^{-3T_2}\right)(\mu_2-\mu_3)}{6(3e^{T_1}-2)}$
L'_D	$\left(\frac{3}{2}e^{-T_2} - \frac{1}{6}e^{-3T_2}\right)(\mu_2-\mu_3) - \frac{4}{3}\mu_2 + 2\mu_3 + T_2\mu_2 + \mu_D T_D$
Δ_D	$\frac{(1-e^{-T_1})(2\mu_2-(3e^{-T_2}-e^{-3T_2})(\mu_2-\mu_3))}{2(3-2e^{-T_1})}$

Balanced

L_I	$\frac{3e^{-T_1}(\mu_1-\mu_3)+\mu_3e^{-(T_1+T_2)}+3e^{-T_2}(\mu_2-\mu_3)+3(T_1\mu_1+T_2\mu_2-\mu_1-\mu_2+2\mu_3)}{3-2e^{-(T_1+T_2)}}$
L'_I	μ_3
Δ_I	$\frac{3(e^{-T_1}(\mu_1-\mu_3)+\mu_3e^{-(T_1+T_2)}+e^{-T_2}(\mu_2-\mu_3)+(T_1\mu_1+T_2\mu_2-\mu_1-\mu_2+\mu_3))}{3-2e^{-(T_1+T_2)}}$
L_A	$\frac{e^{-(T_1+T_2)}(-6T_1\mu_1-7\mu_3)+9((1-e^{-T_1})\mu_1+\mu_3e^{-T_1})}{9-6e^{-(T_1+T_2)}} + \mu_A T_A$
L'_A	$T_1\mu_1 + \frac{2}{3}\mu_3 + \mu_A T_A$
Δ_A	$\frac{-\mu_3e^{-(T_1+T_2)}+3\mu_1(1-e^{-(T_1)}-T_1)+\mu_3(-2+3e^{-(T_1)})}{-2e^{-(T_1+T_2)}+3}$

Table S2: Summary of simplifying assumptions and the corresponding simplified formulas for expected branch lengths in matching and non-matching quartet gene trees. This table is reproduced from Table S2 in Tabatabaee et al. (2023).

Unbalanced

Parameter	Simplified formula	Simplifying assumption
L_I	$\lim_{\mu_3\to\mu_2} L_I = \frac{3\mu_1(e^{-T_1}-1+T_1)}{3-2e^{-T_1}} + \mu_2$	$\mu_3 \to \mu_2$
L'_I	$\lim_{\mu_3\to\mu_2} L'_I = \mu_2$	$\mu_3 \to \mu_2$
Δ_I	$\lim_{\mu_3\to\mu_2} \Delta_I = \frac{3\mu_1(e^{-T_1}-1+T_1)}{3-2e^{-T_1}}$	$\mu_3 \to \mu_2$
L_A	$\lim_{T_2\to\infty} L_A = \frac{6T_1\mu_1+3\mu_1-\mu_2}{6-9e^{T_1}} + \mu_1 + \mu_A T_A$	$T_2 \to \infty$
L'_A	$\lim_{T_2\to\infty} L'_A = \frac{5}{6}\mu_2 + T_1\mu_1 + T_A\mu_A$	$T_2 \to \infty$
Δ_A	$\lim_{T_2\to\infty} \Delta_A = \frac{-6\mu_1\left(e^{-T_1}-1+T_1\right)-(5-4e^{-T_1})\mu_2}{6-4e^{-T_1}}$	$T_2 \to \infty$
L_C	$\lim_{T_2\to\infty} L_C = \mu_2 + \mu_C T_C + \frac{\mu_2}{3(3e^{T_1}-2)}$	$T_2 \to \infty$
L'_C	$\lim_{T_2\to\infty} L'_C = \frac{1}{3}\mu_2 + \mu_C T_C$	$T_2 \to \infty$
Δ_C	$\lim_{T_2\to\infty} \Delta_C = \frac{(2-e^{-T_1})\mu_2}{(3-2e^{-T_1})}$	$T_2 \to \infty$
L_D	$\lim_{\mu_3\to\mu_2} L_D = \mu_2 + T_2\mu_2 + \mu_D T_D - \frac{\mu_2}{3(3e^{T_1}-2)}$	$\mu_3 \to \mu_2$
L'_D	$\lim_{\mu_3\to\mu_2} L'_D = \frac{2}{3}\mu_2 + T_2\mu_2 + \mu_D T_D$	$\mu_3 \to \mu_2$
Δ_D	$\lim_{\mu_3\to\mu_2} \Delta_D = \frac{(1-e^{-T_1})\mu_2}{3-2e^{-T_1}}$	$\mu_3 \to \mu_2$

Balanced

L_I	$\lim_{T_2\to 0} L_I = \frac{3\mu_1(e^{-T_1}-1+T_1)}{3-2e^{-T_1}} + \mu_3$	$T_2 \to 0$
L'_I	μ_3	—
Δ_I	$\lim_{T_2\to 0} \Delta_I = \frac{3\mu_1(e^{-T_1}-1+T_1)}{3-2e^{-T_1}}$	$T_2 \to 0$
L_A	$\lim_{\mu_3\to\mu_1} L_A = \mu_1 + \frac{\mu_1(1+6T_1)}{6-9e^{(T_1+T_2)}} + \mu_A T_A$	$\mu_3 \to \mu_1$
L'_A	$\lim_{\mu_3\to\mu_1} L'_A = T_1\mu_1 + \frac{2}{3}\mu_1 + \mu_A T_A$	$\mu_3 \to \mu_1$
Δ_A	$\lim_{\mu_3\to\mu_1} \Delta_A = \frac{\mu_1\left(-e^{-(T_1+T_2)}+1-3T_1\right)}{-2e^{-(T_1+T_2)}+3}$	$\mu_3 \to \mu_1$

Table S3: Summary of formulas for estimating unbalanced or balanced quartet species tree branch lengths in SU. This table is reproduced from Table S3 in Tabatabaee et al. (2023).

Unbalanced

Parameter	Estimation formula	Simplifying assumption(s)
t_1	$\hat{t_1} = \bar{L}'_I \left(\frac{1}{2}\bar{\delta} + \frac{1}{6}\sqrt{3\bar{\delta}(3\bar{\delta}+4)}\right)$	$\mu_3 \to \mu_2; \mu_1 \to \mu_2$
t_A	$\hat{t_A} = \bar{L}'_A + \frac{\mu_1(e^{-T_1}-1+T_1)+\bar{\Delta}_A(1-2/3e^{-T_1})}{1-4/5e^{-T_1}} - T_1\mu_1$	$T_2 \to \infty$
t_B	$\hat{t_B} = \bar{L}'_B + \frac{\mu_1(e^{-T_1}-1+T_1)+\bar{\Delta}_B(1-2/3e^{-T_1})}{1-4/5e^{-T_1}} - T_1\mu_1$	$T_2 \to \infty$
t_C	$\hat{t_C} = \bar{L}'_C - \frac{1}{3}(2 - \frac{1}{2-e^{-T_1}})\bar{\Delta}_C$	$T_2 \to \infty$
$t_2 + t_D$	$\hat{t_2} + \hat{t_D} = \bar{L}'_D - \frac{2}{3}(2 + \frac{1}{1-e^{-T_1}})\bar{\Delta}_D$	$\mu_3 \to \mu_2$

Balanced

$t_1 + t_2$	$\hat{t_1} + \hat{t_2} = \bar{L}'_I \left(\frac{1}{2}\bar{\delta} + \frac{1}{6}\sqrt{3\bar{\delta}(3\bar{\delta}+4)}\right)$	$T_2 \to 0; \mu_1 \to \mu_3$
t_A	$\hat{t_A} = \bar{L}'_A - \frac{2}{3}\mu_1 - \frac{1}{3}\left(\mu_1\left(1 - e^{-(T_1+T_2)}\right) - \bar{\Delta}_A\left(3 - 2e^{-(T_1+T_2)}\right)\right)$	$\mu_3 \to \mu_1$
t_B	$\hat{t_B} = \bar{L}'_B - \frac{2}{3}\mu_1 - \frac{1}{3}\left(\mu_1\left(1 - e^{-(T_1+T_2)}\right) - \bar{\Delta}_B\left(3 - 2e^{-(T_1+T_2)}\right)\right)$	$\mu_3 \to \mu_1$
t_C	$\hat{t_C} = \bar{L}'_C - \frac{2}{3}\mu_2 - \frac{1}{3}\left(\mu_2\left(1 - e^{-(T_1+T_2)}\right) - \bar{\Delta}_C\left(3 - 2e^{-(T_1+T_2)}\right)\right)$	$\mu_3 \to \mu_2$
t_D	$\hat{t_D} = \bar{L}'_D - \frac{2}{3}\mu_2 - \frac{1}{3}\left(\mu_2\left(1 - e^{-(T_1+T_2)}\right) - \bar{\Delta}_D\left(3 - 2e^{-(T_1+T_2)}\right)\right)$	$\mu_3 \to \mu_2$

S3 Details of the Experimental Study

S3.1 Simulated Datasets

ILS-only dataset. We reused a published dataset from Tabatabaee et al. (2023) simulated using Simphy. This dataset has 50 repliates, each with 100 ingroups and one outgroup species and 1000 gene trees. In addition to true gene trees, gene alignments of length 1600bp, 800bp, 400bp, and 200bp were simulated under GTR+Γ, and four sets of gene trees were estimated from these alignments using FastTree-2 (Price et al., 2010), producing gene trees with 23%, 31%, 42%, and 55% GTEE. Measuring ILS using the average Robinson and Foulds (1981) (RF) distance (AD for short) between the model species tree and true gene trees, ILS is heterogeneous across replicates and ranges from 30% to 58%, with a mean of 46% AD.

GDL+ILS. We updated the Simphy-generated GDL+ILS datasets of Willson et al. (2022, 2023) to have species trees with SU branch lengths. Here, locus trees evolve inside the species tree with GDL events only; the final true gene trees evolve on the locus tree under MSC. Therefore, the topological differences between the final true gene trees and the locus tree are only due to ILS; we use the normalized RF distance between the locus tree and the true gene trees to measure ILS. This dataset has model conditions (10 replicates each) characterized by two levels of ILS (low and high ILS with 20% and 65% average ILS level, respectively), six duplication rates, ranging from 10^{-13} to 10^{-9}, three sequence lengths (50bp, 100bp, 500bp), four different numbers of species (21, 51, 101, or 1001), and five numbers of genes (50, 100, 500, 1000, 10,000). The loss rate varies based on the duplication rate, with three different ratios: 1 (equal loss), 0.5 or 0 (no loss). In the default model condition, the loss rate is equal to the duplication rate. Gene trees were estimated using FastTree-2. The number of replicates in all model conditions is 10. Table S4 summarizes further statistics about the model conditions of this dataset.

HGT+ILS. We recreated a 50-replicate 51-taxon (50 ingroup and one outgroup) dataset with both HGT and ILS based on the parameters used by Davidson et al. (2015). The ILS level is fixed at 30% AD across the model conditions. The six model conditions differ in HGT rates, leading to total discordance that varies between 30% to 68%. The average number of HGT events per gene for the six model conditions starts from 0 and increases to 0.08, 0.2, 0.8, 8 and 20 that correspond to HGT rates of 0, 2×10^{-9}, 5×10^{-9}, 2×10^{-8}, 2×10^{-7} and 5×10^{-7}. In addition to true gene trees, we simulated 1000bp gene sequence alignments using INDELible (Fletcher and Yang, 2009) under the GTR+Γ model and then used FastTree-2 to estimate gene trees under the GTR model. GTEE is on average 28% and about the same in all model conditions. The number of genes is 1000 and the number of replicates is 50. Table S5 summarizes the empirical statistics of this dataset.

Table S4: Empirical statistics of the simulated GDL datasets. AD refers to average RF distance between the locus tree and the true gene trees, and GTEE refers to average RF distance between true and estimated gene trees. L/D refers to the ratio between loss and duplication rates. The last column shows the average number of leaves in each gene family tree across the replicates. Default parameters: 1000 genes, 100bp sequence length, 10 replicates, 1 L/D ratio.

Dup. rate	# Taxa	AD	GTEE (500bp)	GTEE (100bp)	GTEE (50bp)	L/D ratio	# Leaves
Low ILS							
1×10^{-13}	21	18.4%	–	37.9%	–	1	21.0
1×10^{-12}	21	22.1%	18.4%	41.5%	52.5%	1	21.0
1×10^{-12}	51	20.9%	–	42.1%	–	1	51.0
1×10^{-12}	101	25.1%	–	45.9%	–	1	101.2
1×10^{-11}	21	25.1%	–	42.2%	–	1	21.3
1×10^{-10}	21	19.0%	–	36.8%	–	1	24.1
1×10^{-10}	101	23.4%	–	44.0%	–	1	116.6
1×10^{-10}	101	23.5%	–	45.0%	–	0.5	128.0
1×10^{-10}	101	24.0%	–	44.1%	–	0	145.1
5×10^{-10}	21	15.8%	–	34.2%	–	1	35.8
5×10^{-10}	101	20.3%	19.29%	43.36%	55.68%	1	165.3
5×10^{-10}	101	22.71%	–	45.08%	–	0.5	290.6
5×10^{-10}	101	26.59%	–	43.09%	–	0	550.0
5×10^{-10}	1001	23.49%	–	44.4%	–	1	1578.1
1×10^{-9}	21	15.6%	–	33.7%	–	1	52.1
1×10^{-9}	101	19.1%	–	39.7%	–	1	228.5
1×10^{-9}	101	20.2%	–	43.4%	–	0.5	993.0
1×10^{-9}	101	23.9%	–	47.2%	–	0	3727.8
High ILS							
1×10^{-13}	21	69.3%	–	42.2%	–	1	21.0
1×10^{-12}	21	67.4%	19.2%	42.6%	55.7%	1	21.0
1×10^{-12}	51	75.7%	–	45.8%	–	1	51.2
1×10^{-12}	101	78.4%	–	48.2%	–	1	101.2
1×10^{-11}	21	67.0%	–	41.0%	–	1	21.3
1×10^{-10}	21	64.5%	–	39.0%	–	1	24.2
5×10^{-10}	21	54.5%	–	39.5%	–	1	36.2
5×10^{-10}	101	50.0%	–	43.9%	–	1	170.1
1×10^{-9}	21	44.2%	–	38.3%	–	1	47.9

Table S5: Empirical statistics of the simulated HGT datasets. AD refers to average RF distance between the model species tree and true gene trees, and GTEE refers to average RF distance between true and estimated gene trees. The number of taxa is 51 and the number of genes is 1000.

HGT rate	Expected number of HGT events per gene	AD	GTEE
0	0	29.7%	27.0%
2×10^{-9}	0.08	30.1%	31.2%
5×10^{-9}	0.2	30.9%	28.9%
2×10^{-8}	0.8	34.1%	28.4%
2×10^{-7}	8	53.4%	27.0%
5×10^{-7}	20	68.4%	26.9%

S3.2 Biological Datasets

Birds. We studied the birds dataset of Stiller et al. (2024) including 363 species and 63,430 genes that was used to resolve family-level relationships among neoavian species and is expected to have high levels of ILS due to a rapid radiation. The original study had inferred the tree topology using ASTRAL and then estimated branch lenghts on that topology using the concatenation of all 63K genes. We infer branch lengths on the same ASTRAL topology using CASTLES-Pro.

Bees. We renalyzed the bees dataset of Bossert et al. (2021) containing 32 species (30 ingroups and two outgroups) from the bee subfamily Nomiinae and 853 gene trees (estimated using RAxML). We used the ASTRAL topology from the original study, and used partitioned concatenation (with RAxML) and CASTLES-Pro to draw branch lengths on this topology.

Mammals. We studied the mammalian biological dataset from Song et al. (2012), including 37 species (36 ingroup and 1 outgroup) and 447 gene trees, which was reduced to 424 trees after removing gene trees with mis-matching names (Mirarab et al., 2014). We estimated an ASTRAL topology and estimated branch lengths using concatenation and CASTLES-Pro on that topology.

Fungi. We examined the fungal dataset of Butler et al. (2009), including 16 yeast species and 7,180 multi-copy gene family trees. The original study had used MrBayes (Huelsenbeck and Ronquist, 2001) on a concatenated alignment created by sampling 30,000 sites from 706 individual gene family orthologous peptide sequences. We used ASTRAL-Pro2 (Zhang and Mirarab, 2022) to estimate a species tree using all 7,180 gene family trees, and used CASTLES-Pro to estimate branch lengths on that tree. The two trees are different in one branch, with an RF distance of 7.6%.

Plants (1KP). We analyzed the plants dataset of Wickett et al. (2014), that included 103 species and 424 single-copy gene trees, as well as 9,610 multi-copy gene family trees for 83 of the species, that was left unsued in the original study due to lack of propor method for estimating the species tree from multi-copy input. The gene trees were inferred using RAxML for the first two codon positions (C12) in the transcriptome. We compare the branch lengths of the concatenation tree inferred from the 424 single-copy gene alignments with a tree inferred using ASTRAL-Pro2 from all 9,610 multi-copy gene trees furnished with CASTLES-Pro branch lengths. The two trees have 80 taxa in common and are different in 7 branches on the shared set of taxa, resulting in an RF distance of 9.1%.

Eudicots. We studied the 40-taxon angiosperm dataset of Chanderbali et al. (2022) focused on the Eudicots lineage. This study had used three sets of genes to perform phylogenomic analysis using concatenation and coalescent-based summary methods: 345 filetered single-copy Angiosperms353 loci (Johnson et al., 2019), 1248 single-copy BUSCO (Simão et al., 2015) genes, and 2,573 multi-copy orthogroups. The authors had performed concatenation analysis on the two sets of single-copy genes (Angiosperms353 loci and BUSCOs) using RAxML and coalescent analysis on all three sets of genes using ASTRAL and ASTRAL-Pro for single and multi-copy input respectively. We compared the two concatenation trees from the original study with a tree we inferred using ASTRAL-Pro2 from the 2,573 orthogroups that was furnished with CASTLES-Pro branch lengths. The two concatenation trees had the same topology that was different from the ASTRAL-Pro2 tree in three branches, with an RF distance of 8.1%.

Microbial datasets. We analyzed three microbial datasets including thousands of species of bacteria and archaea and different sets of genes to study a debate about the length of the branch separating domains archaea and bacteria (AB branch). While the long-standing hypothesis was that these two domains are separated by a long branch (Gogarten et al., 1989; Iwabe et al., 1989; Cox et al., 2008), a recent study (Zhu et al., 2019) had estimated a far shorter length for the AB branch than what was previously expected using a concatenation analysis. Moody et al. (2022) had further studied this and other bacterial datasets, and suggested that concatenation can severaly underestimate branch lengths on datasets with high levels of HGT, resulting in short estimates of Zhu et al. (2019).

Here we examine two bacterial datasets analyzed by Moody et al. (2022) and the Web of Life (WoL) dataset from Zhu et al. (2019) with CASTLES-Pro to further study this debate. The two bacterial datasets include a 72-taxon dataset with 49 core genes originally from Williams et al. (2020) that includes ribosomal proteins and other conserved elements and a 108-taxon dataset with 38 genes from Petitjean et al. (2015) that only includes non-ribosomal proteins. The WoL dataset included 10,575 species (9,906

bacteria and 669 archaea) and 381 marker genes including ribosomal and non-ribosomal proteins. For the two smaller bacterial datasets, we estimated a species tree using ASTRAL on the two gene sets and used concatenation (with RAxML) and CASTLES-Pro to draw branch lengths on these topologies. On the WoL dataset, we used the ASTRAL tree from the original study that was furnished with branch lengths estimated using RAxML from a concatenation including 100 sites randomly selected from sites with less than 50% gaps for each marker gene. We estimated branch lengths on the same topology using CASTLES-Pro.

Table S6: Empirical statistics of the biological datasets. ILS, GDL and HGT refer to the main source of gene tree discordance in each dataset.

Study	Dataset type/species	# taxa	# single-copy genes	# multi-copy genes
		**ILS**		
Stiller et al. (2024)	Neoavian birds	363	63,430	NA
Song et al. (2012)	Mammals	37	424	NA
Bossert et al. (2021)	Bees (subfamily Nomiinae)	32	853	NA
		**GDL**		
Wickett et al. (2014)	Plants (1kp)	80	424	9,610
Chanderbali et al. (2022)	Eudicots	40	345	2,573
Butler et al. (2009)	Fungi	16	706	7,180
		**HGT**		
Williams et al. (2020)	Bacterial (core genes)	72	49	NA
Petitjean et al. (2015)	Bacterial (non-ribosomal genes)	108	38	NA
Zhu et al. (2019)	Bacterial (WoL)	10,575	381	NA

S3.3 Methods and Software Commands

Here we bring the details of the methods and software commands. All experiments were performed on the University of Illinois campus cluster, with a memory limit of 128GB.

- **DISCO.** We used DISCO (Willson et al., 2022) version 1.3 to decompose multi-copy gene-family trees into single-copy gene trees. DISCO is available at https://github.com/JSdoubleL/DISCO. We used the following command:

```
python3 disco.py -i <multi-copy-gene-trees> -o <single-copy-gene-trees> -d _
```

- **CA-DISCO.** To run concatenation on multi-copy gene family sequences, we used the script ca_disco.py available at https://github.com/JSdoubleL/DISCO with the following command:

```
ca_disco.py -i <gene_tree_path> -a <alignment_list_path> -t <taxa_list_path>
-o <output_path> -d _
```

  where <gene_tree_path> is the path to the set of multi-copy gene family trees, <alignment_list_path> is a file containing the list of individual sequence alignments for each gene family, and <taxa_list_path> is the set of all taxa. The output is the concatenated sequence alignment, that is then passed to RAxML (v8.2.12) (Stamatakis, 2014), available at https://github.com/stamatak/standard-RAxML, to optimize branch lengths on a fixed tree topology with the option -f e using the following command.

```
raxmlHPC -PTHREADS -f e -t <species_tree_path> -m GTRGAMMA -s <alignment_path>
-n RES -p 4321 -T 16
```

- **ERaBLE.** To run ERaBLE (Binet et al., 2016), we first calculated a matrix of pairwise patristic distances per gene using a custom script available at https://github.com/ytabatabaee/CASTLES-Pro-paper/scripts/patristic_dist_matrix.py that uses calculate.treecompare._get_length_diffs from the package DendroPy (Sukumaran and Holder, 2010). When calculating the patristic distance matrix, we impute missing values with averages: i.e., if two taxa i and j do not appear in the same gene tree g together due to missing taxa in genes, as is the case in DISCO gene trees, the patristic distance of i and j in the matrix for gene tree g is replaced by the average patristic distances of these two taxa in the rest of the gene trees where they appear together. We used the following command to run this script

```
python3 patristic_dist_matrix.py -g <gene_tree_path> -o <dist_mat.phylip>
-m all
```

  Then we ran ERaBLE (v1.0) available at http://www.atgc-montpellier.fr/erable/ with the following command;

```
erable -i <dist_mat.phylip> -t <species_tree_path> -o <output_path>
```

- **FastME.** Similar to ERaBLE, to run FastME (Lefort et al., 2015), we first computed a *single* distance matrix corresponding to average patristic distances between pairs of taxa using the following command

```
python3 patristic_dist_matrix.py -g <gene_tree_path> -o <dist_mat.phylip>
-m avg
```

  and then we ran FastME version 2.1.6.2 with the following command

```
fastme-2.1.6.2-linux64 -i <dist_mat.phylip> -w BalLS -u <species_tree_path>
-o <output_path>
```

- **CASTLES-Pro.** CASTLES-Pro is integrated inside the species tree estimation software ASTER that is available at https://github.com/chaoszhang/ASTER. To infer branch lengths on a fixed tree topology using ASTER (v1.19.3.5), we used the following commands for multi-copy and single-copy gene trees respectively

```
bin/astral-pro2 -i <gene-tree-path> -C -c <species-tree-topology> -o <output-path>
--root <outgroup-name> --genelength <gene-sequence-length>
```

```
bin/astral4 -i <gene-tree-path> -C -c <species-tree-topology> -o <output-path>
--root <outgroup-name> --genelength <gene-sequence-length>
```

where `--root` specifies the outgroup name (if known) and `--genelength` specifies the average gene sequence length (default: 1000bp).

- **CASTLES.** CASTLES (Tabatabaee et al., 2023) is available at https://github.com/ytabatabaee/CASTLES. To run it, we first annotated the species tree toplogy using ASTER with the following command

  ```
  astral -C -i <gene-tree-path> -c <species-tree-topology> -o <output_path>
  --root <outgroup-name> > <annotated.tre>
  ```

  where the annotated tree is printed to the file `<annotated.tre>`. We then ran the CASTLES script (v1.0) using the following command

  ```
  python3 castles.py -t <annotated.tre> -g <gene_tree_path> -o <output_path>
  ```

- **TCMM.** TCMM (Arasti et al., 2024) is available at https://github.com/shayesteh99/TCMM. To run the per-gene version, we used the command:

  ```
  python3 multiple_tree_matching.py -i <species-tree-topology> -r <gene_tree_path>
  -l <lambda> -o <output_path>
  ```

  To run the consensus version of TCMM, we used the command:

  ```
  python3 weighted_tree_matching.py -i <species-tree-topology> -r <gene_tree_path>
  -l <lambda> -o <output_path>
  ```

S4 Additional Results (Figures and Tables)

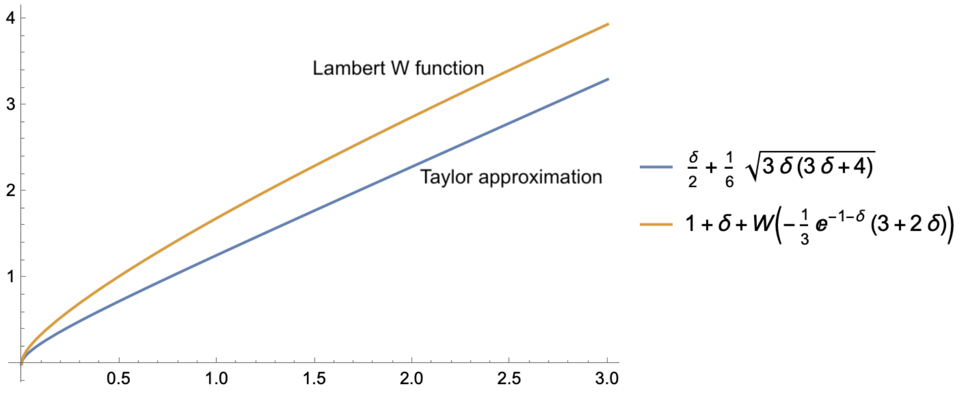

Figure S4: Lambert W function vs its Taylor approximation for caluclating the length of the internal branch $g(\bar{\delta})$ in CASTLES-Pro for $\delta \in (0, 3)$.

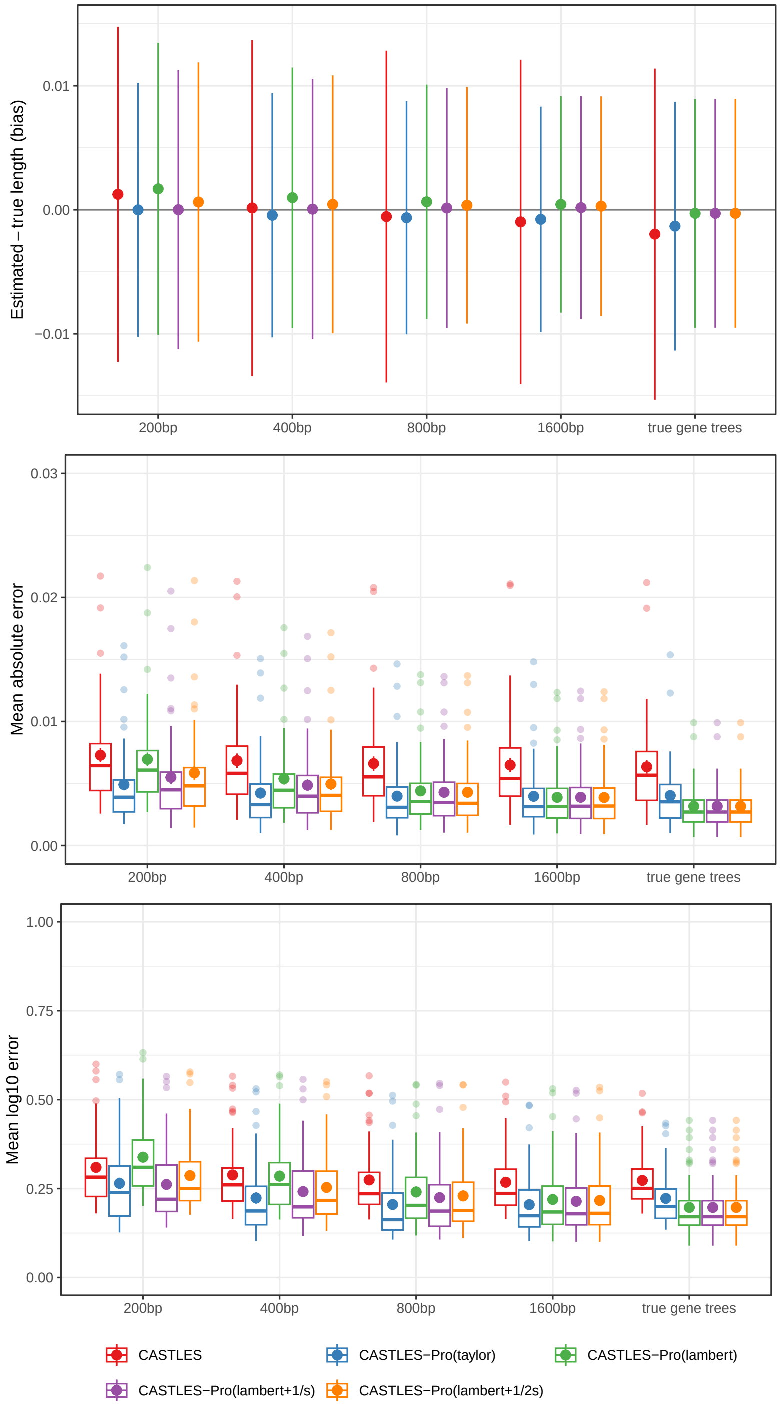

Figure S5: Bias, mean absolute error and mean log error for variants of CASTLES-Pro and CASTLES on 100-taxon simulated ILS datasets. The four variants of CASTLES-Pro either use the taylor approximation for calculating the length of the internal branch, or lambert function with two different pseudo-counts based on sequence lengths. The average ILS level on this dataset is 47% AD and the GTEE level varies between 0% for true gene trees to 55% for gene trees estimated from 200bp alignments. The number of genes is 1000 and the number of replicates is 50. The method shown in purple (`CASTLES-Pro(lambert+1/s)`) is the variant that we selected. The difference between CASTLES and CASTLES–Pro(taylor) is the way they compute mutation rates (see Supplementary Section S2).

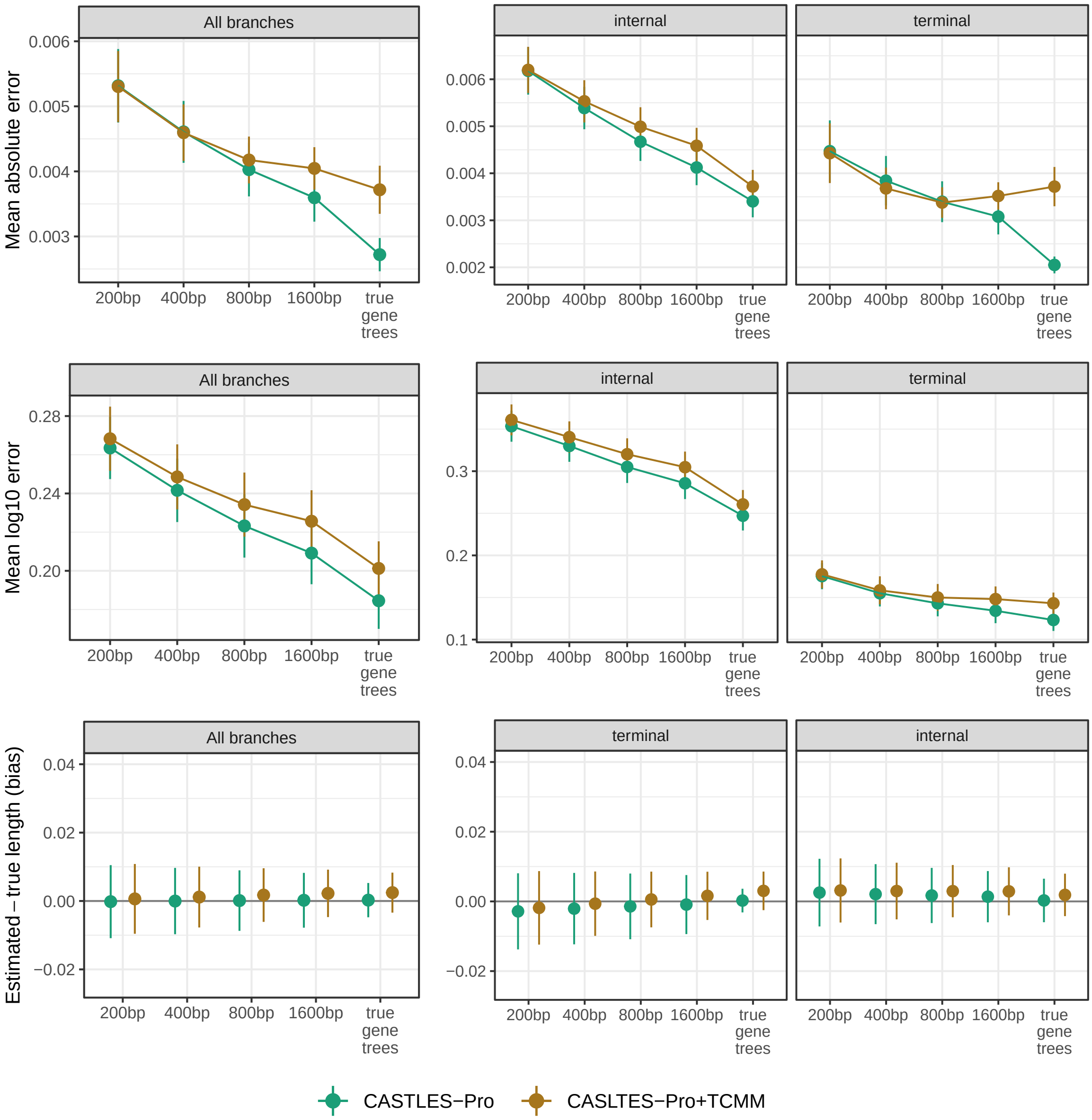

Figure S6: Bias, mean absolute error and mean log error for CASTLES-Pro and CASTLES-Pro+TCMM on 100-taxon simulated ILS datasets. The average ILS level on this dataset is 47% AD and the GTEE level varies between 0% for true gene trees to 55% for gene trees estimated from 200bp alignments. The number of genes is 1000 and the number of replicates is 50.

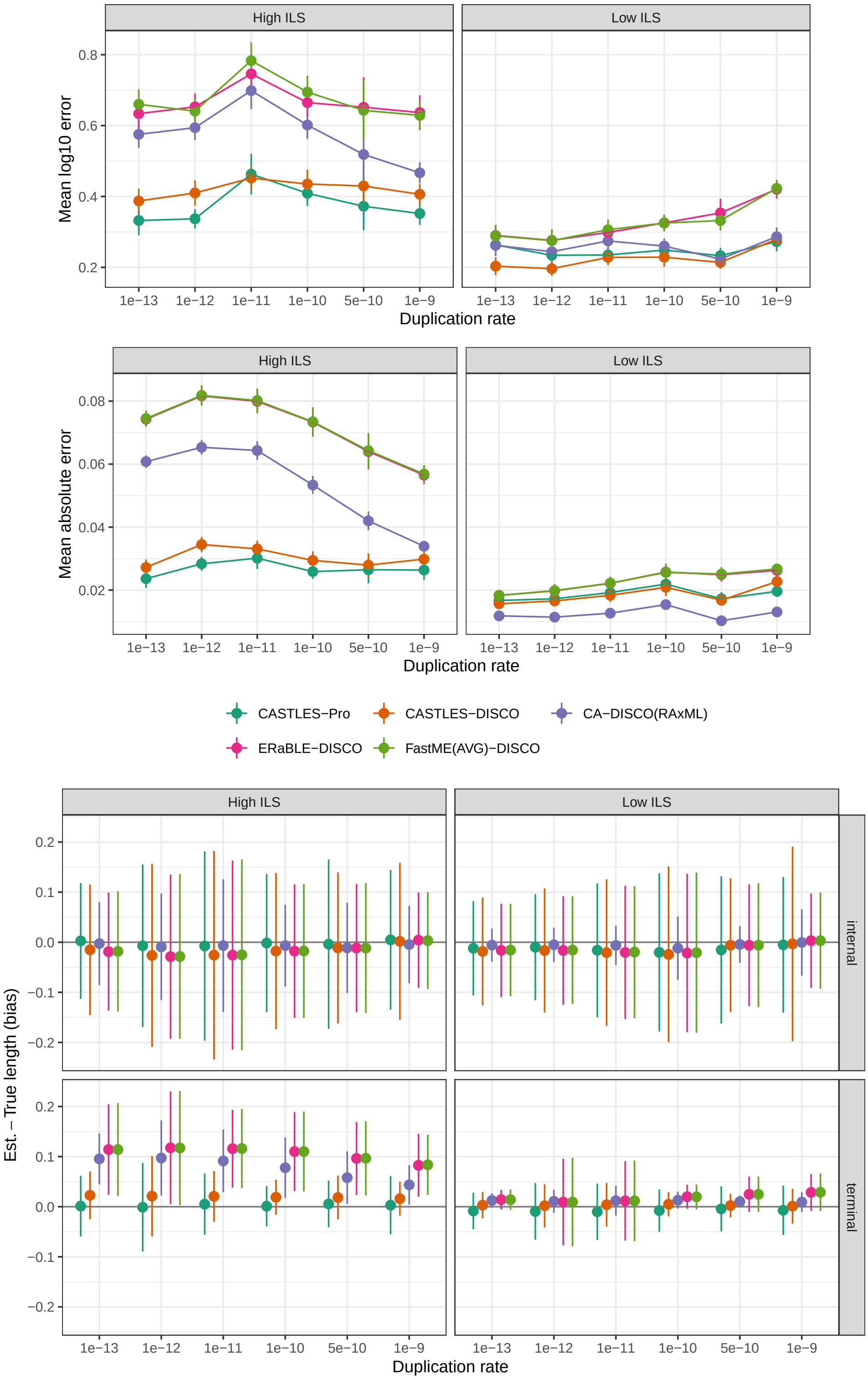

Figure S7: Mean log error, mean absolute error and bias on the GDL datasets for varying duplication rates. The gene trees are estiamted from 100bp alignments with average GTEE levels that varies between 33.7% to 42.2% for the low ILS condition and 38.3% to 42.6% for the high ILS condition. The number of taxa is 20, the number of genes is 1000 and the number of replicates is 10.

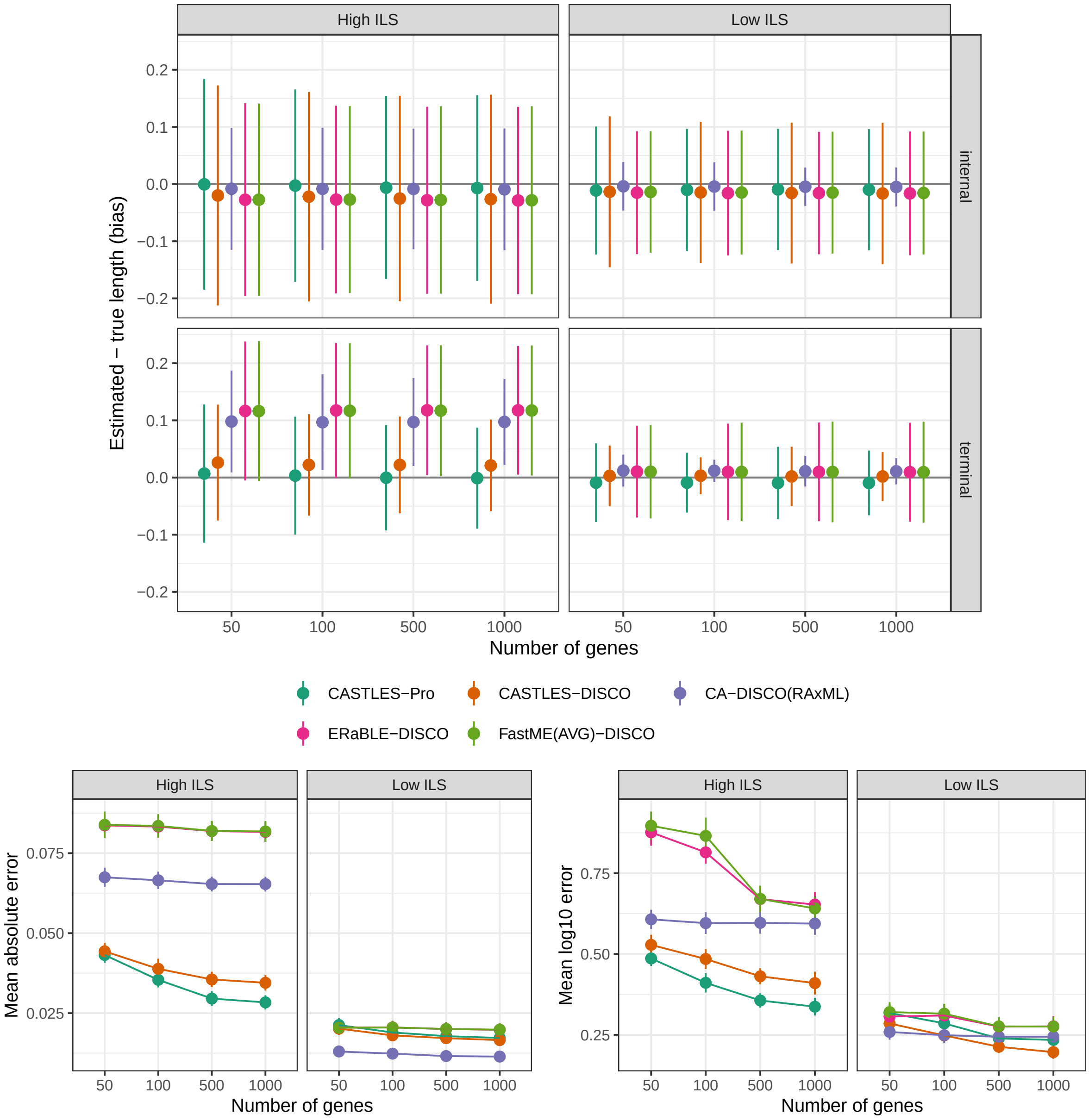

Figure S8: Bias, mean absolute error and mean log error for simulated GDL datasets for varying number of genes. The duplication rate is 10^{-12} with an equal loss rate. Gene trees are estiamted from 100bp alignments and the average GTEE rates for 1000 genes for the low ILS and high ILS conditions are 41.5% and 42.6% respectively. The number of taxa is 20 and the number of replicates is 10.

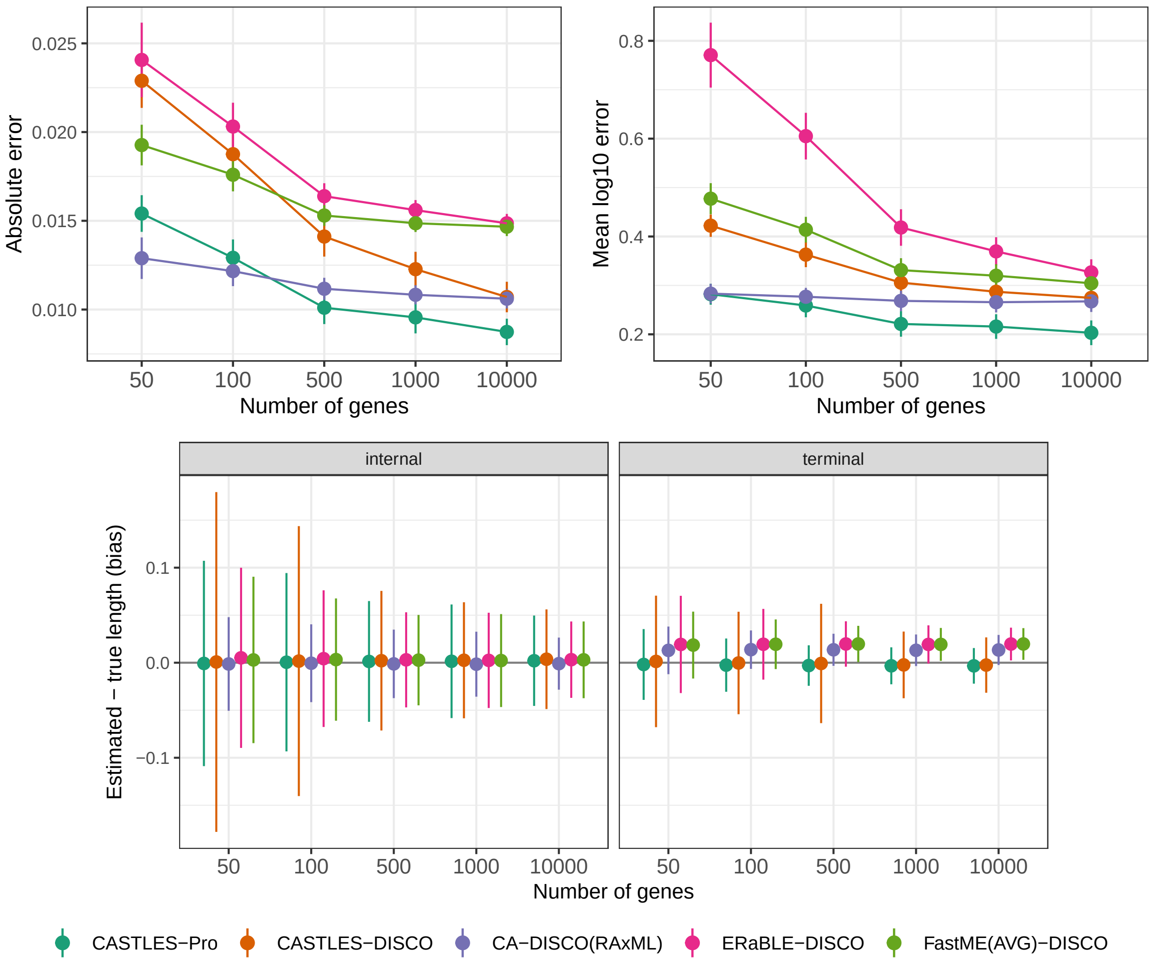

Figure S9: Mean log error, mean absolute error and bias on the 100-taxon GDL datasets for different number of genes. The duplication rate is 5×10^{-10} with equal loss rate and the level of ILS is low (20.3% AD). Gene trees are estiamted from 100bp alignments with an average GTEE level of 41.1% for 10,000 genes. The number of replicates is 10.

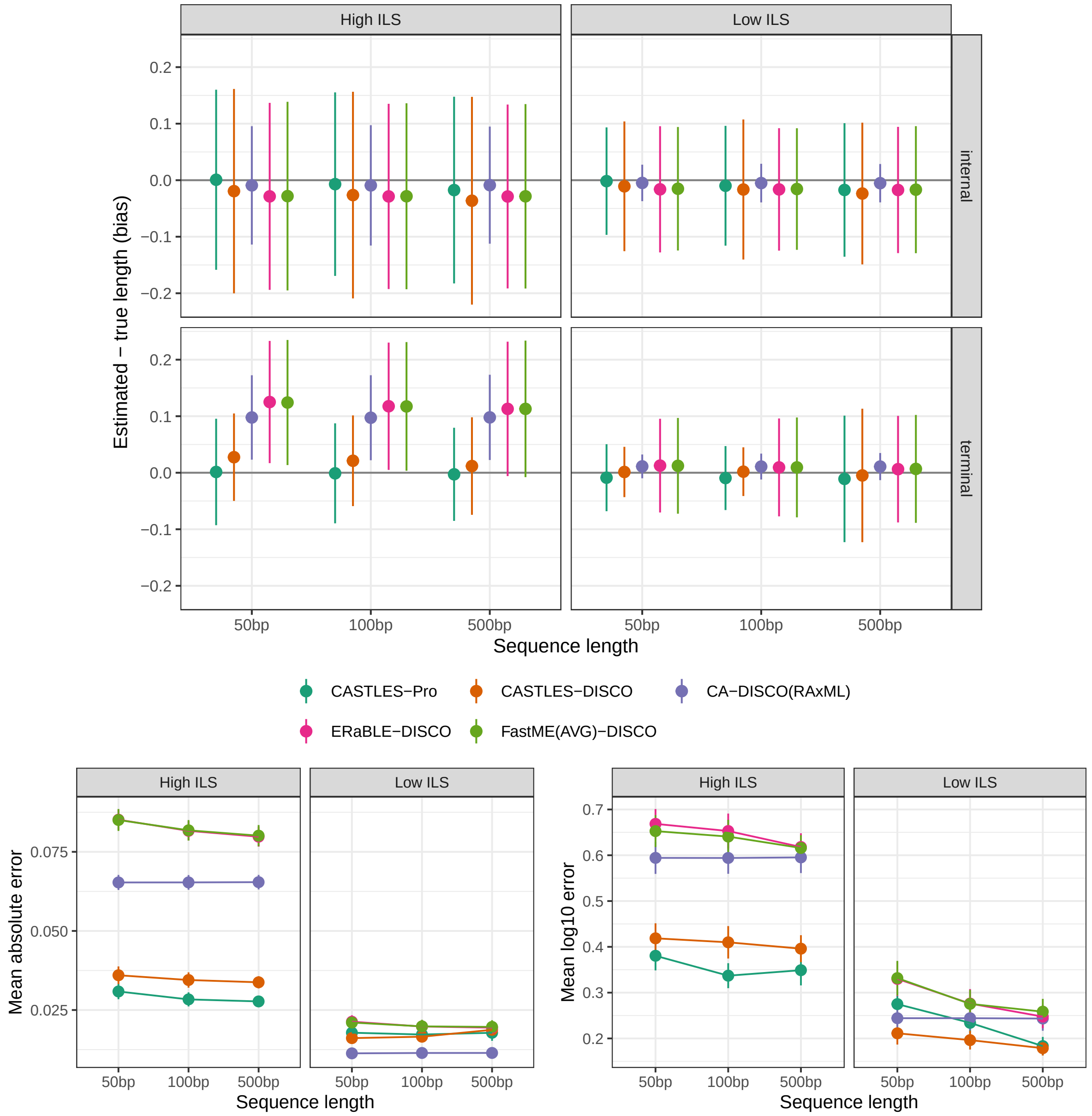

Figure S10: Bias, mean absolute error and mean log error for simulated GDL datasets for varying sequence lengths. The duplication rate is 10^{-12} with an equal loss rate. The average GTEE rates for the 50bp, 100bp and 500bp alignments for the low ILS condition are 52.5%, 41.5% and 18.4% respectively and for the high ILS condition are 55.7%, 42.6% and 19.2%. The number of taxa is 20, the number of genes is 1000 and the number of replicates is 10.

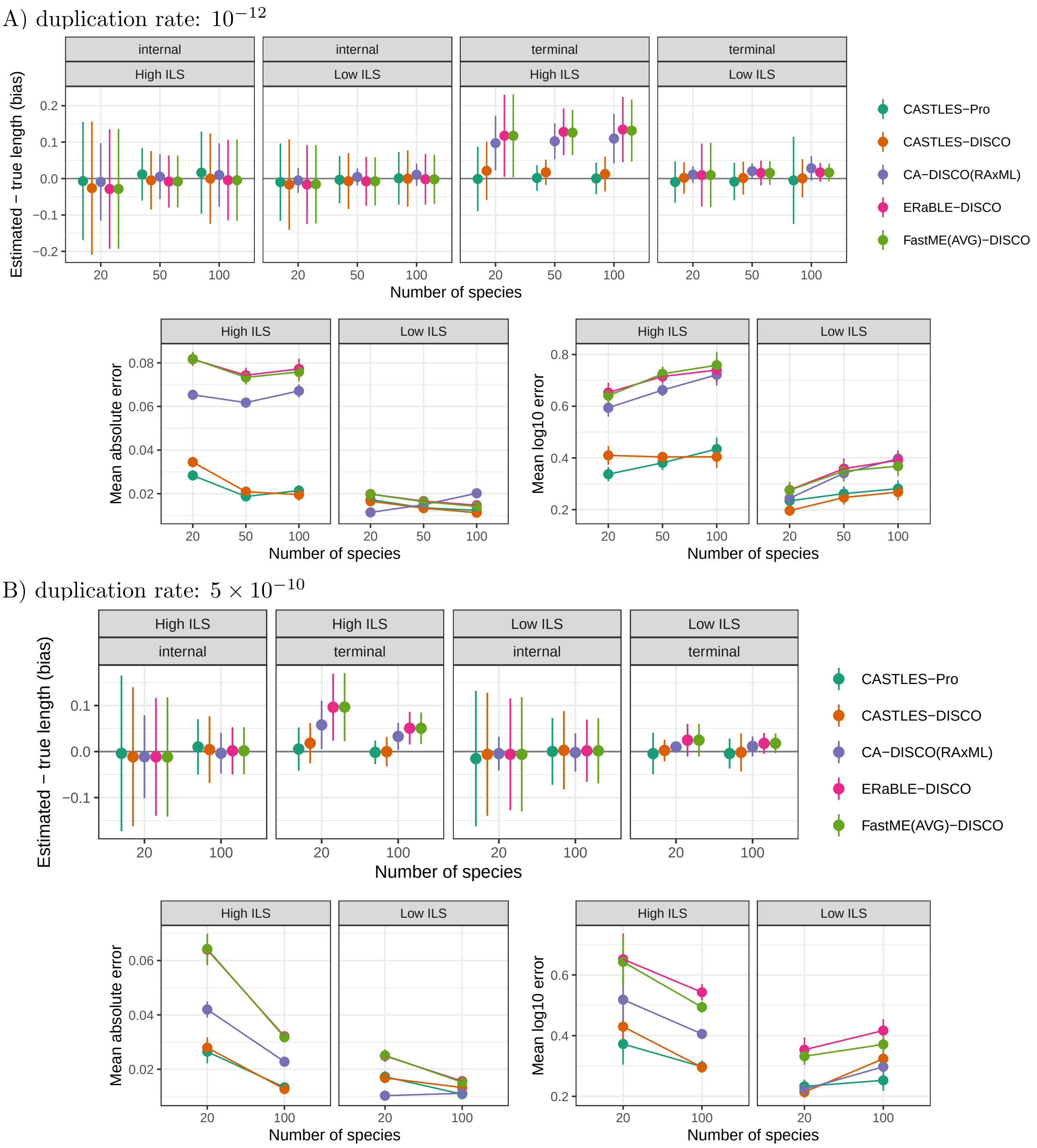

Figure S11: Bias, mean absolute error, and mean log error for simulated GDL datasets for varying numbers of species and level of ILS. The duplication rate is 10^{-12} (A) or 5×10^{-10} (B) with an equal loss rate. The gene trees are estimated from 100bp alignments with an average GTEE level that varies between 34.2% to 48.2% for different conditions. The number of genes is 1000, and the number of replicates is 10.

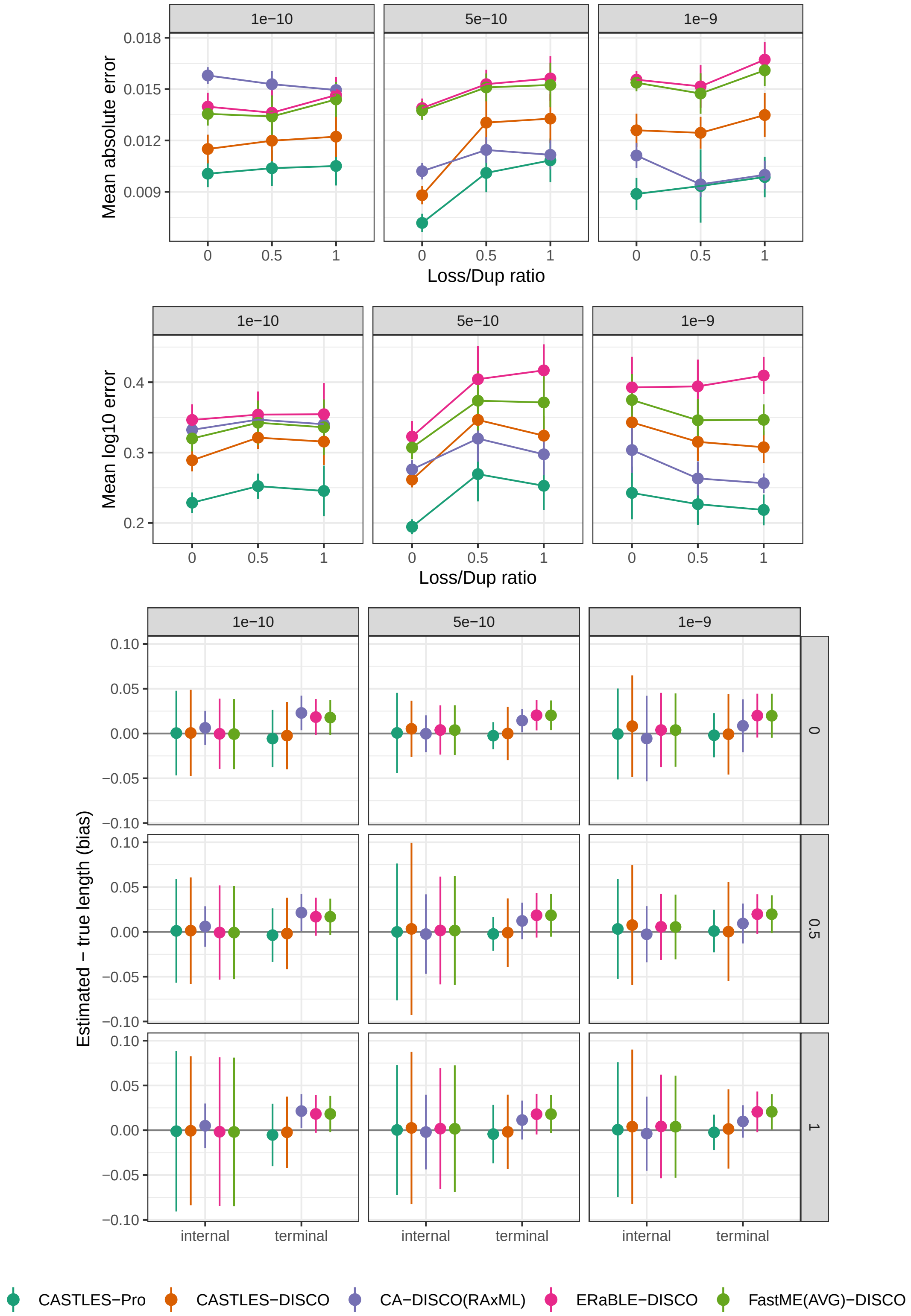

Figure S12: Mean log error, mean absolute error and bias on the 100-taxon GDL datasets for varying duplication/loss ratios. The gene trees are estiamted from 100bp alignments with average GTEE levels that varies between 39.7% to 47.2%. The level of ILS varies between 19.1% to 26.6%. The number of taxa is 100, the number of genes is 1000 and the number of replicates is 10.

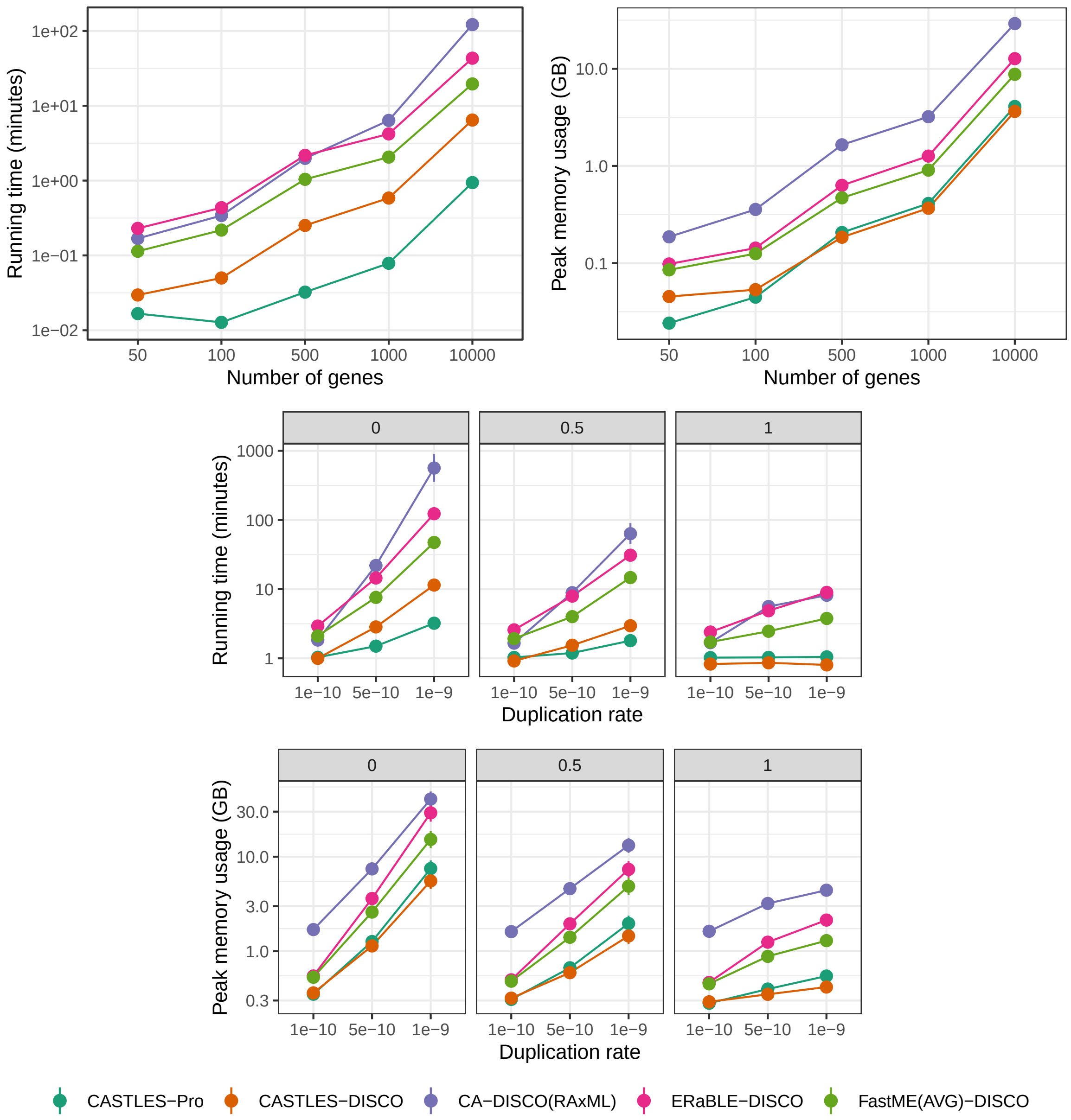

Figure S13: Runtime and peak memory usage of branch length estimation methods on 100-taxon GDL datasets for different number of genes and duplication rates. Gene trees are estimated from 100bp sequence alignments. The y-axes are shown in log scale. (top) The duplication rate is 5×10^{-10} with equal loss rate and the number of genes varies between 50 to 10,000. (bottom) The duplication rate varies between 10^{-10} to 10^{-9} and the number of genes is 1000. The panels show L/D ratio. The number of replicates is 10. The runtime does not include gene tree estimation or species tree topology estimation time, as all methods draw branch lengths on a fixed species tree topology.

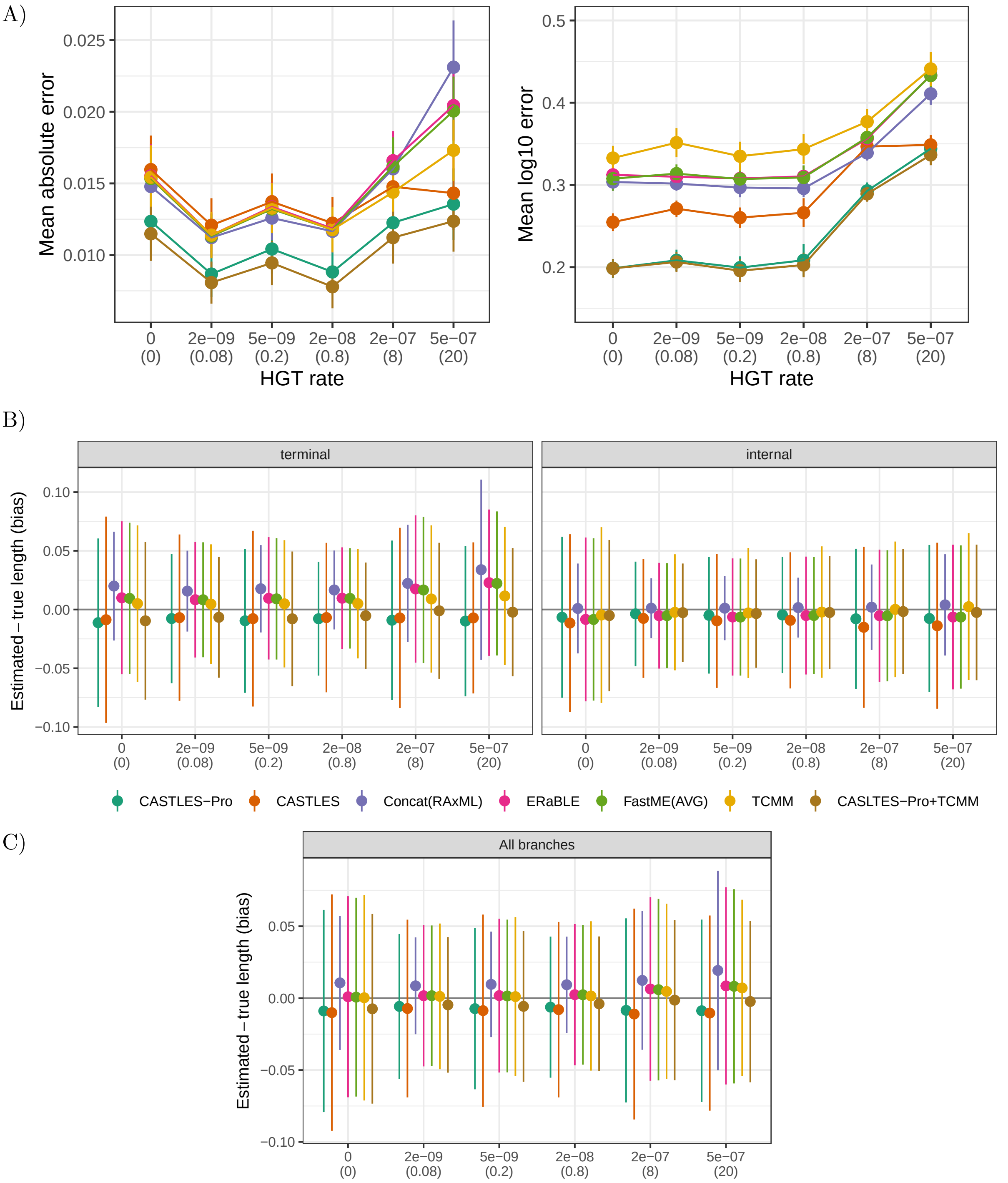

Figure S14: Mean absolute error, mean log error, and bias of branch length estimation methods on 50-taxon simulated HGT datasets. The x-axis indicates the rate of HGT and the expected number of HGT events per gene (in parentheses). The number of replicates is 50, and the number of genes is 1000.

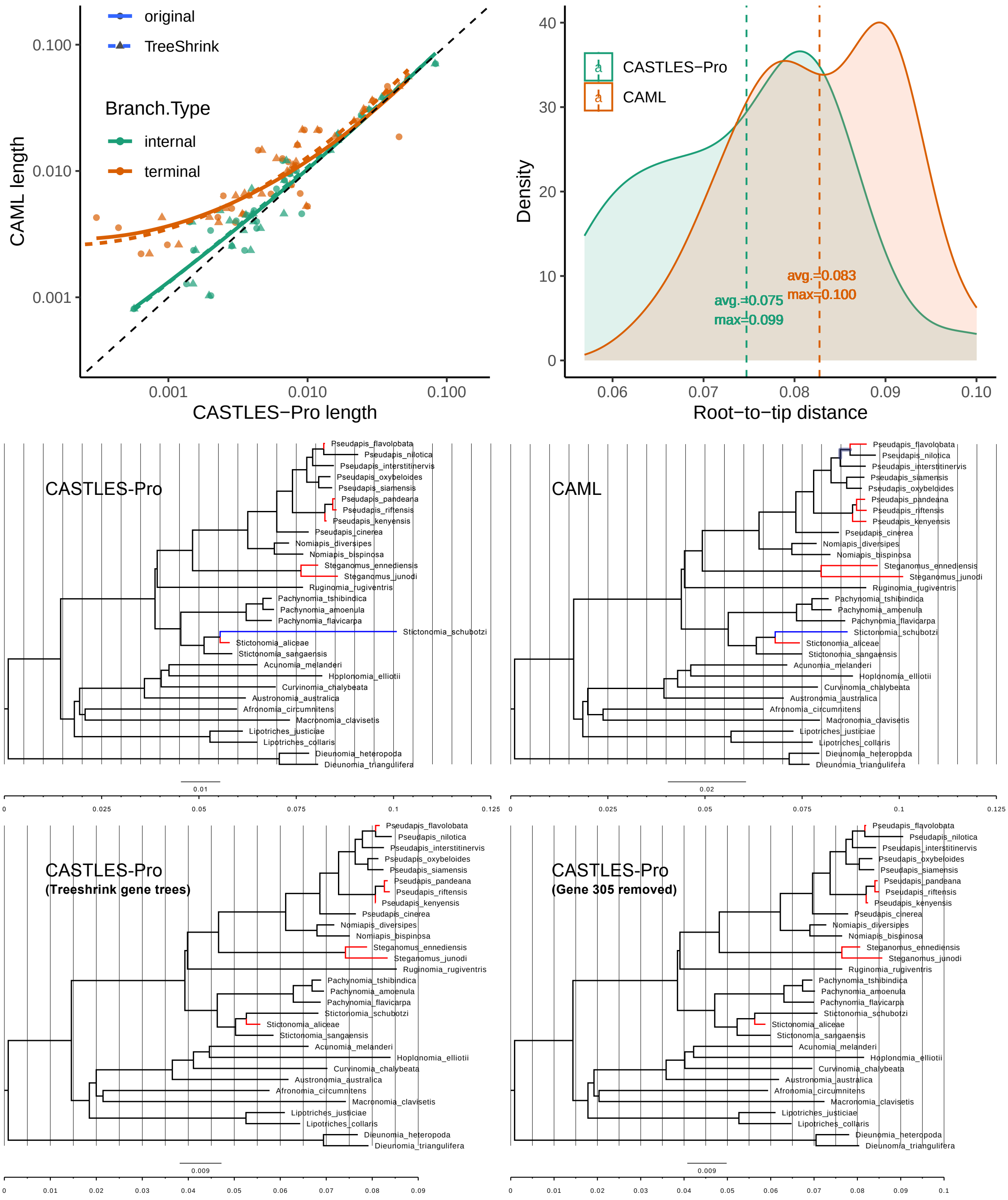

Figure S15: Comparison between the branch lengths of CASTLES-Pro (with or without TreeShrink) and CAML on the bees dataset of Bossert et al. (2021) after removing the outgroup taxa *Lasioglossum albipes* and *Dufourea novaeanglia*. The number of species (after removing the outgroups) is 30 and the number of gene trees is 853. We used the ASTRAL topology from the original study, and used concatenation and CASTLES-Pro to draw branch lengths on this topology. We set the average gene sequence length in CASTLES-Pro as 650bp, as the concatenated alignment had 867 loci with 576,041bp length in total, with an average loci length of 664.4. The branch lengths that are at least 2x longer or shorter in CASTLES-Pro tree compared to the concatenation tree are highlighted in blue and red respectively. The CASTLES-Pro tree estimates an unusually long branch length for taxon *Stictonomia schubotzi* due to an outlier gene, that is fixed when using TreeShrink gene trees or gene trees with the outlier gene 305 removed.

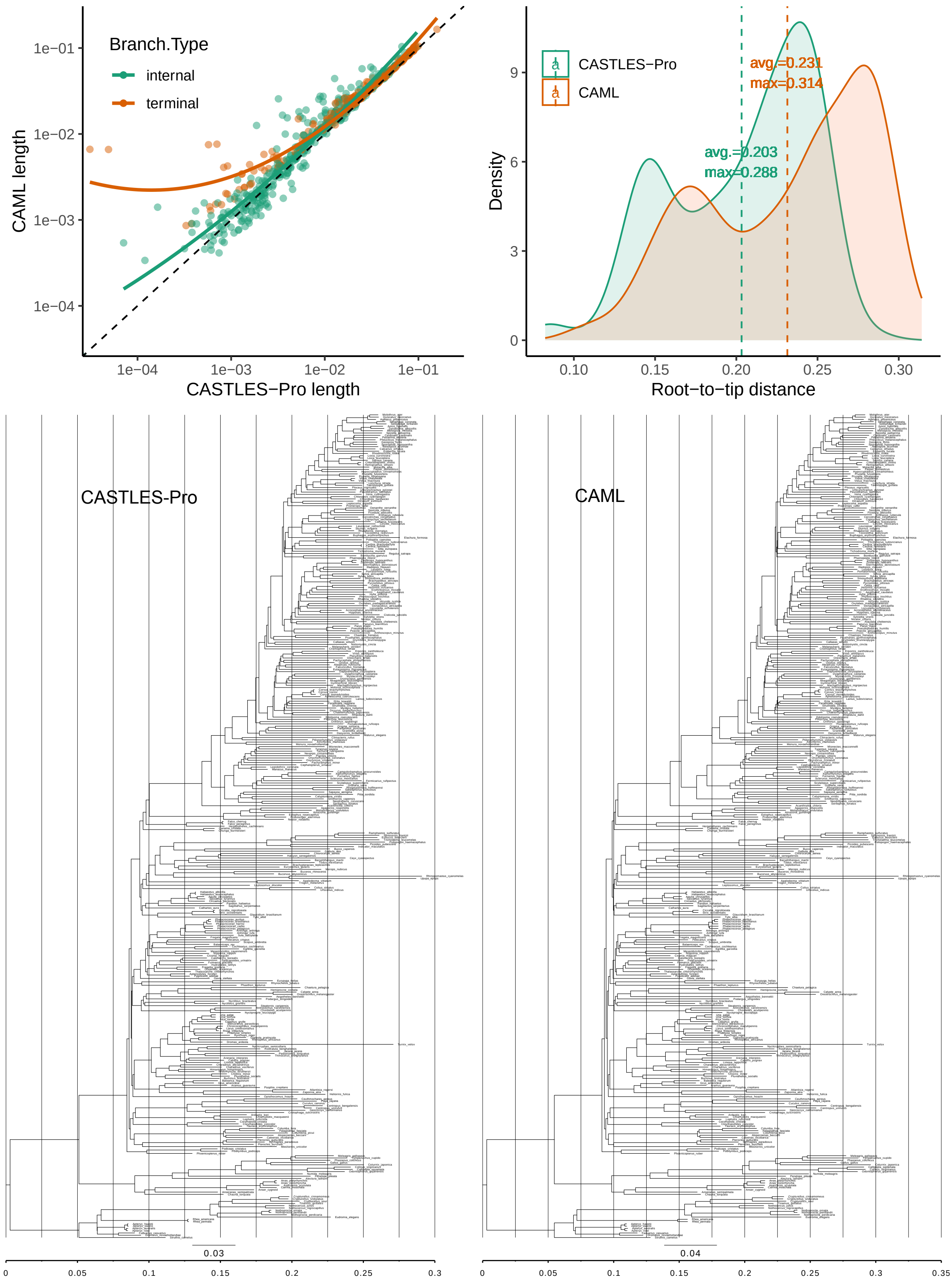

Figure S16: Comparison between branch lengths of CASTLES-Pro and CAML on the birds datasets of Stiller et al. (2024). The number of species is 363 and the number of gene trees is 63,430. We used the ASTRAL topology from the original study with concatenation branch lengths, and estimated branch lengths with CASTLES-Pro on the same topology.

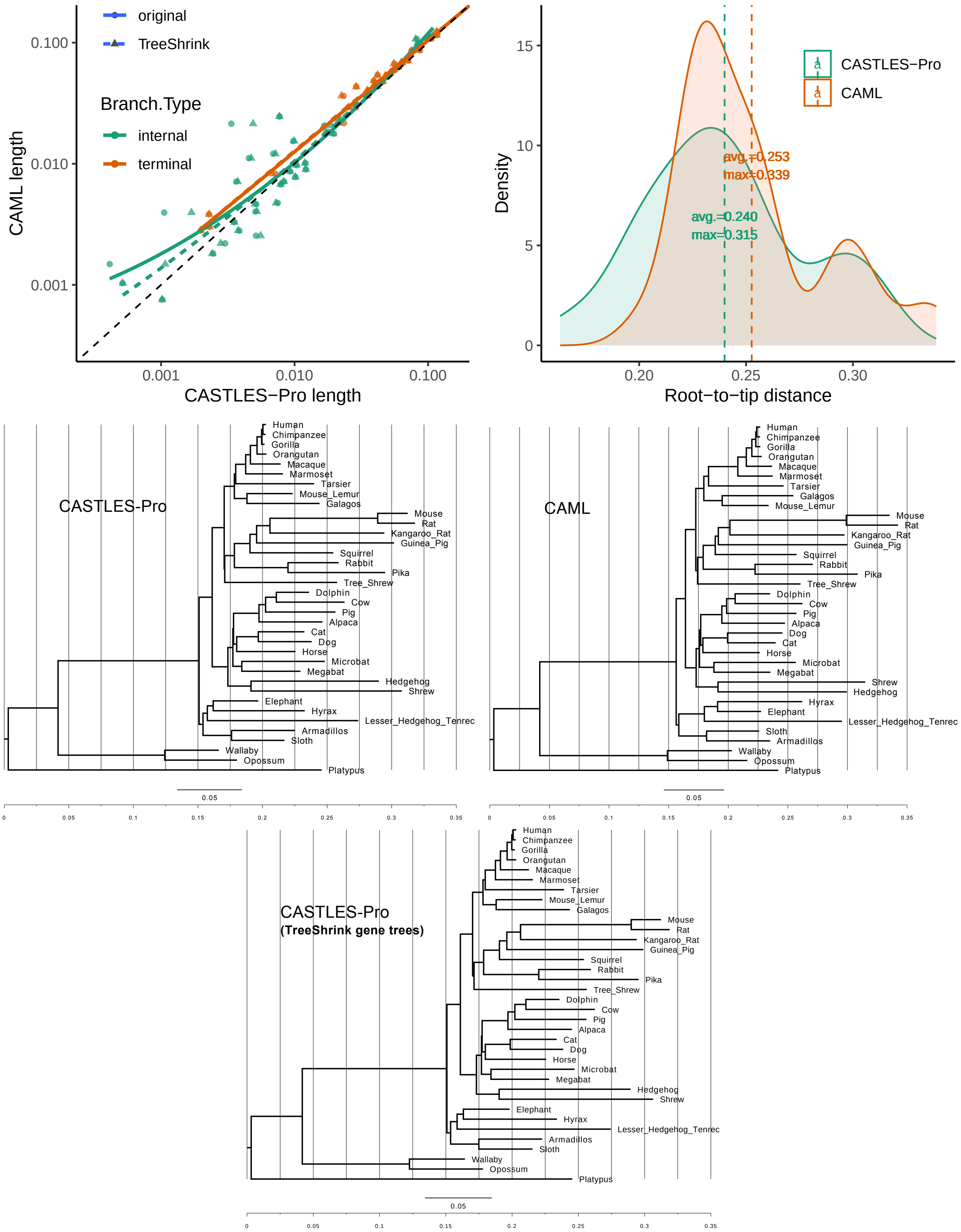

Figure S17: Comparison between branch lengths of CASTLES-Pro (with or without TreeShrink) and CAML on the mammals datasets of Song et al. (2012). The number of species is 37 and the number of gene trees is 424. The average gene sequence length is 3099bp. We draw branch lengths using CASTLES-Pro and concatenation on an ASTRAL species tree topology. The branch lengths are drawn after removing the outgroup taxa *Chicken*.

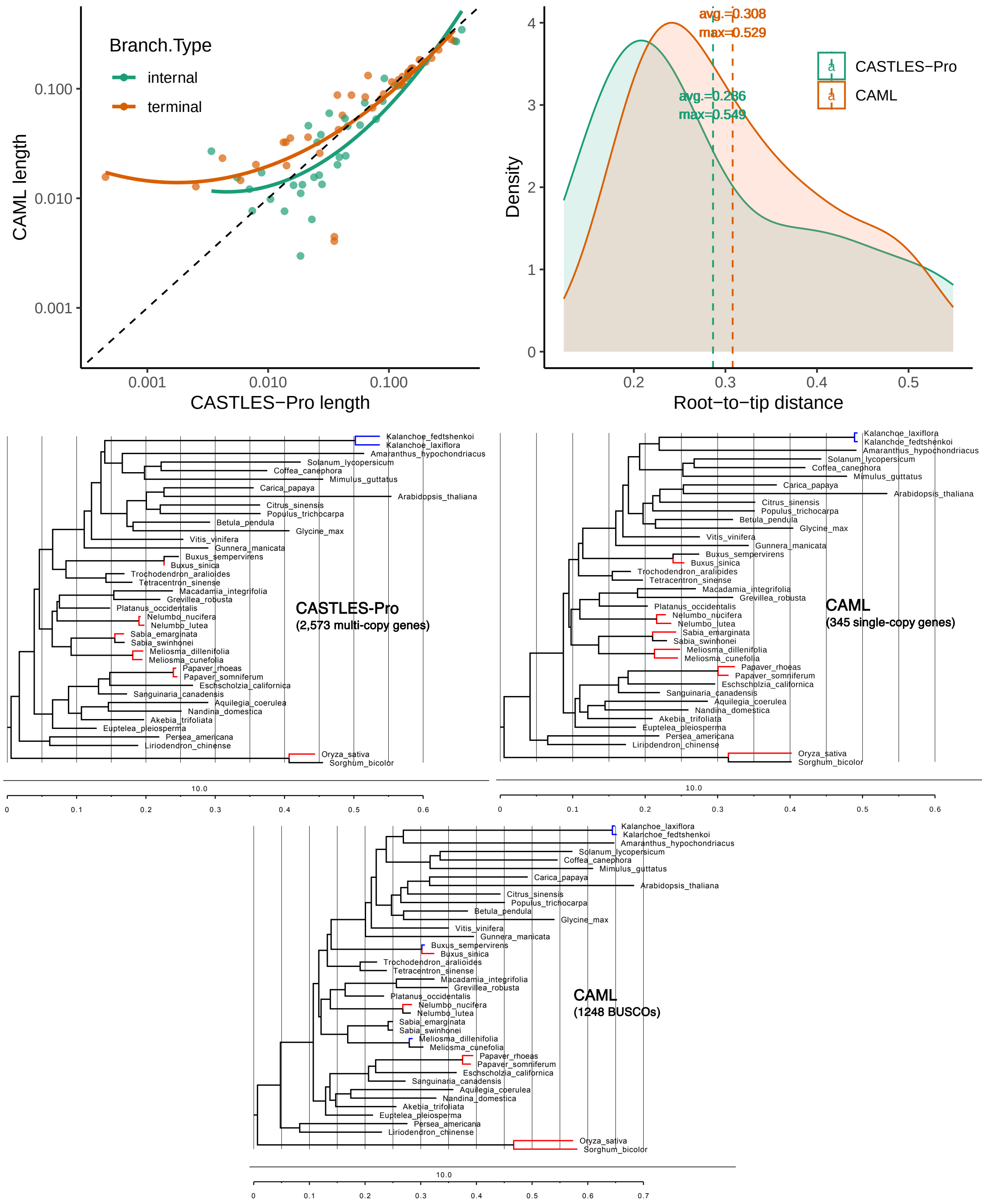

Figure S18: Comparison between branch lengths of CASTLES-Pro and CAML based on Angioseprm353 genes and BUSCO genes on the 40-taxon Eudicots datasets of Chanderbali et al. (2022) (after removing the outgroup *Amborella trichopoda*). The two CAML trees are estimated from the concatenation of 345 filtered Angioseprm353 single-copy genes or 1248 BUSCO single-copy genes, and CASTLES-Pro uses the 2,753 multi-copy gene family trees. The coalescent tree is estimated using ASTRAL-Pro2 and is different in 3 branches with the two concatenation trees (which are identical). The branch lengths that are at least 2x longer or shorter in CASTLES-Pro tree compared to the concatenation trees are highlighted in blue and red respectively.

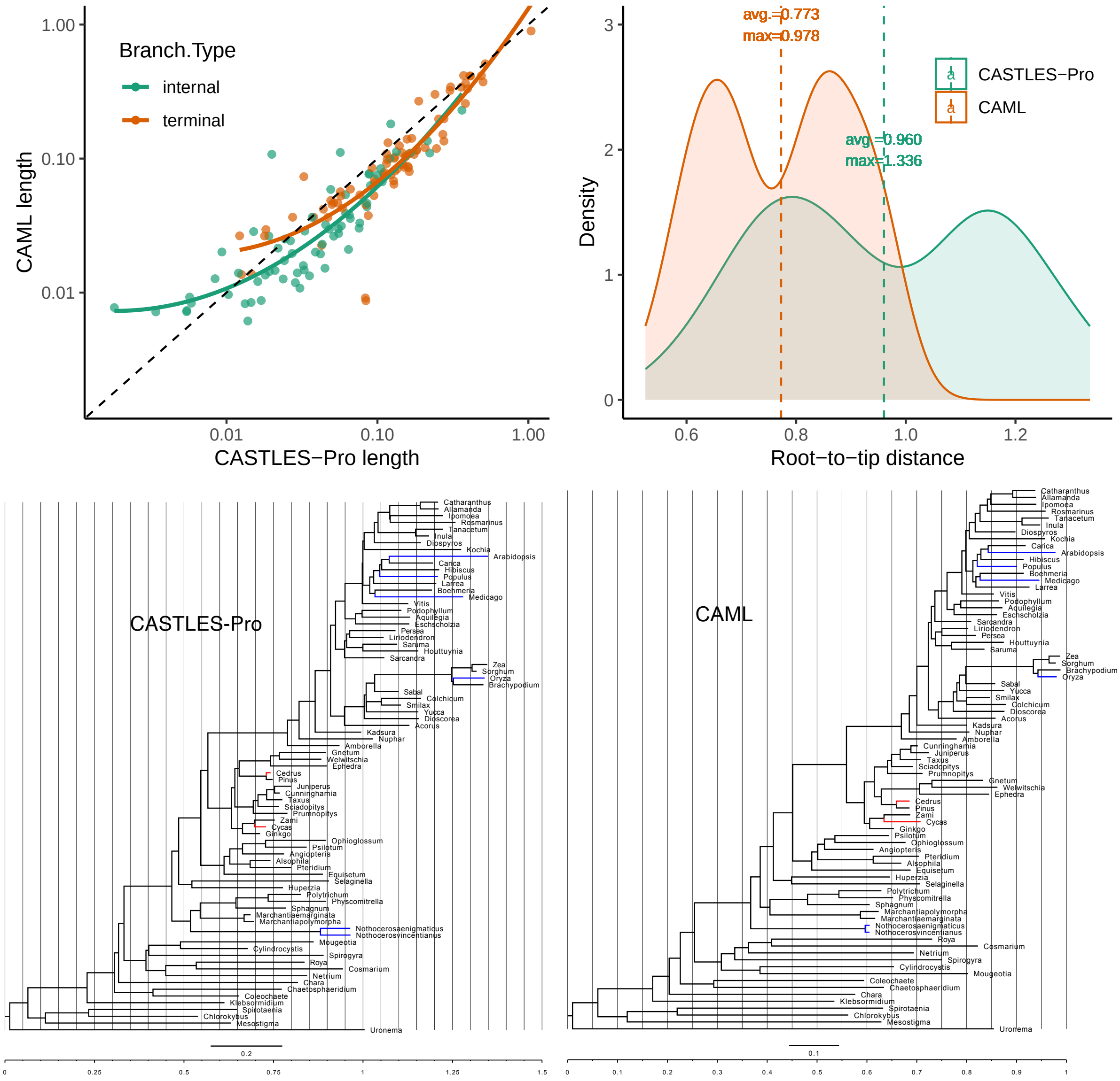

Figure S19: Comparison between the branch lengths of CASTLES-Pro and CAML on the plants dataset of Wickett et al. (2014). The CAML tree is from the original study and was estimated from the concatenated alignment of 424 single-copy genes. In addition, we estimated a tree from the set of 9,610 multi-copy gene family trees from that study using ASTRAL-Pro2 and used CASTLES-Pro to draw branch lengths on this tree. The taxa in the two sets of genes are not entirely identical (see Wickett et al. (2014) for more detail). The RF distance between the CAML and ASTRAL-Pro trees on 79 shared taxa is 9.2%. The correlation is reported only for the shared branches between two trees. The branch lengths that are at least 2x longer or shorter in CASTLES-Pro tree compared to the concatenation tree are highlighted in blue and red respectively.

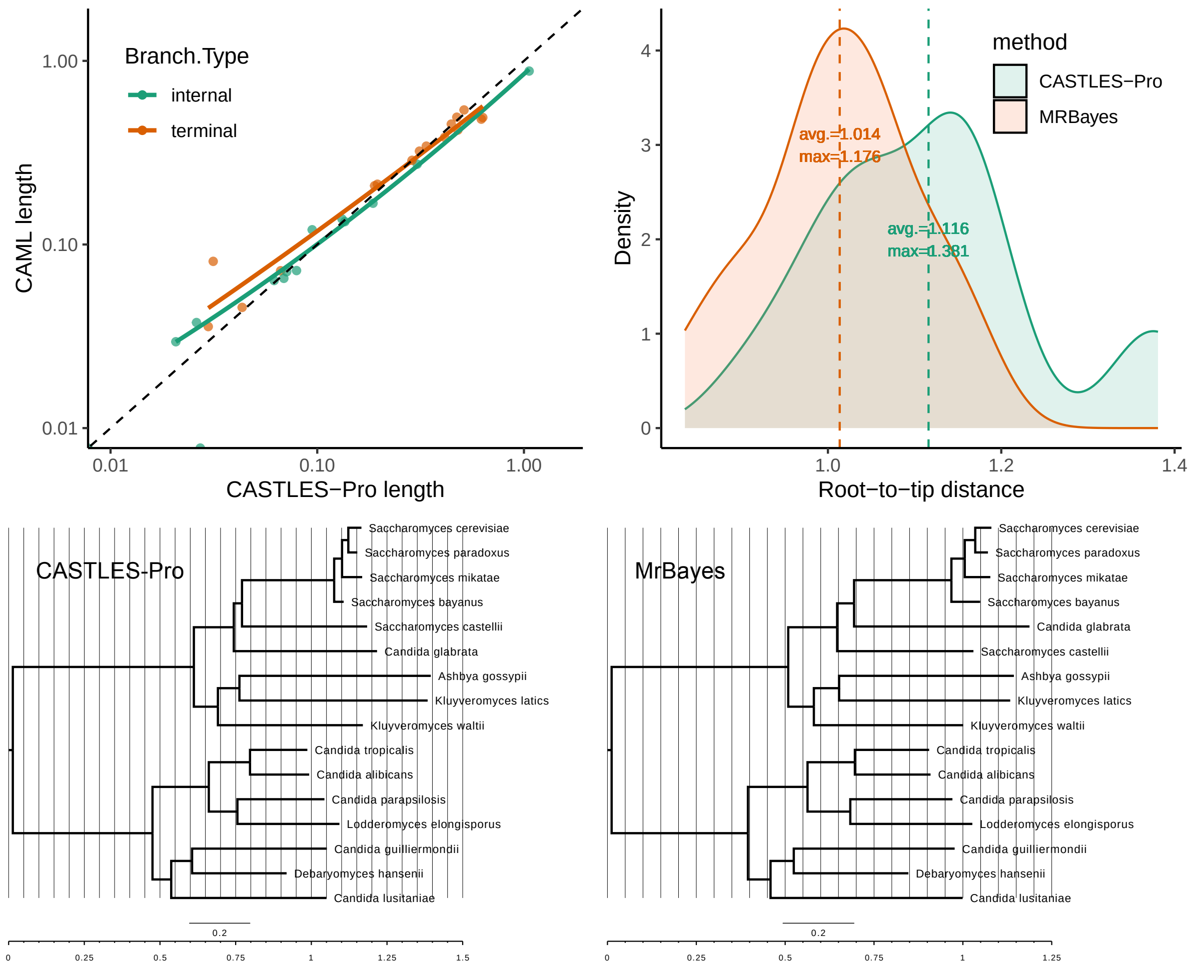

Figure S20: Comparison between branch lengths of CASTLES-Pro and concatenation with MrBayes on the fungal datasets of Butler et al. (2009). The number of species is 16. The original study had used MrBayes (Huelsenbeck and Ronquist, 2001) on a concatenated alignment created by sampling 30,000 sites from 706 individual gene family orthologous peptide sequences. We used ASTRAL-Pro2 to estimate a species tree using all 7,180 gene family trees, and used CASTLES-Pro to estimate branch lengths on that tree. The two trees are different in one branch, with an RF distance of 7.6%.

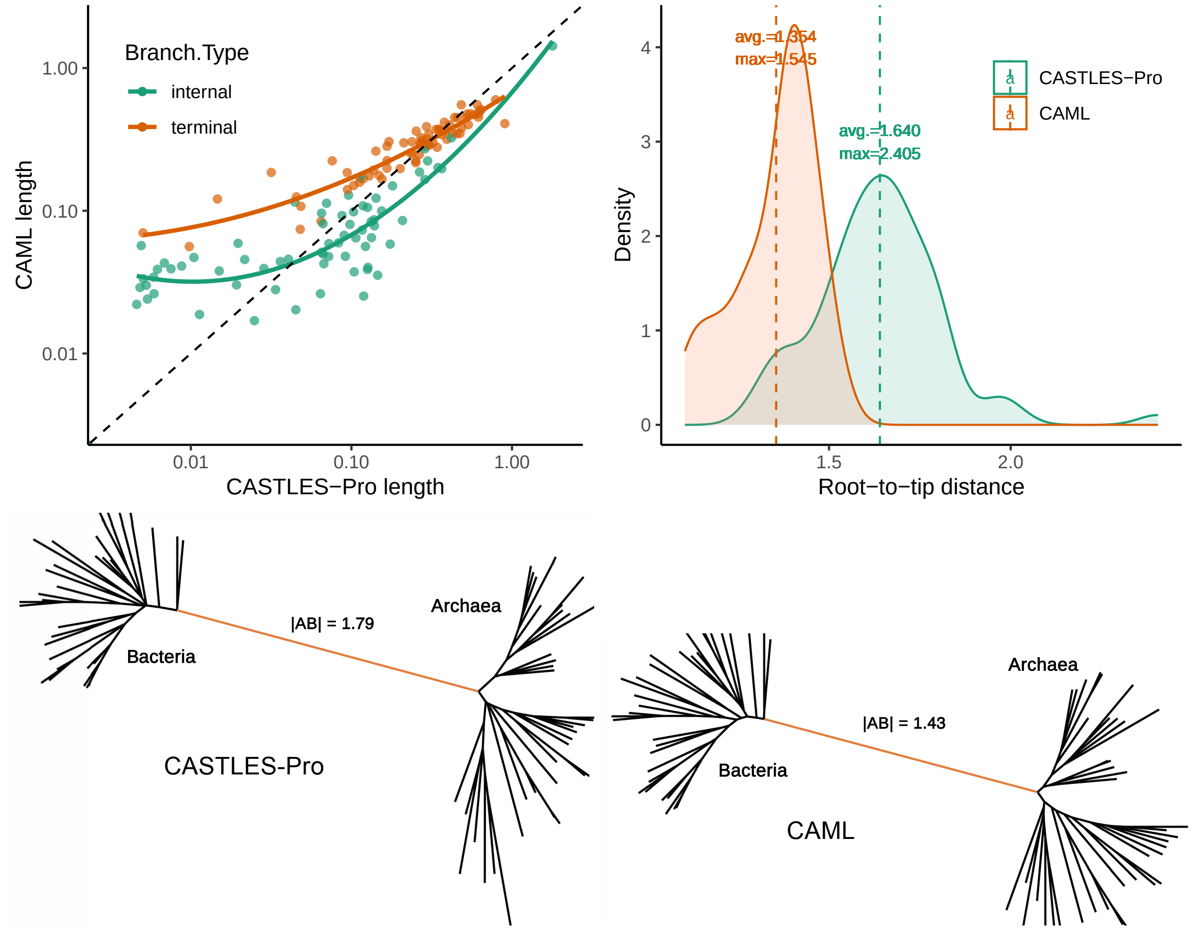

Figure S21: Comparison between branch lengths of CASTLES-Pro and CAML on the bacterial dataset with core genes from Williams et al. (2020). The number of species is 72 and the number of genes is 49. Both methods draw branch lengths on an ASTRAL tree topology. The branch colored in orange is the AB branch.

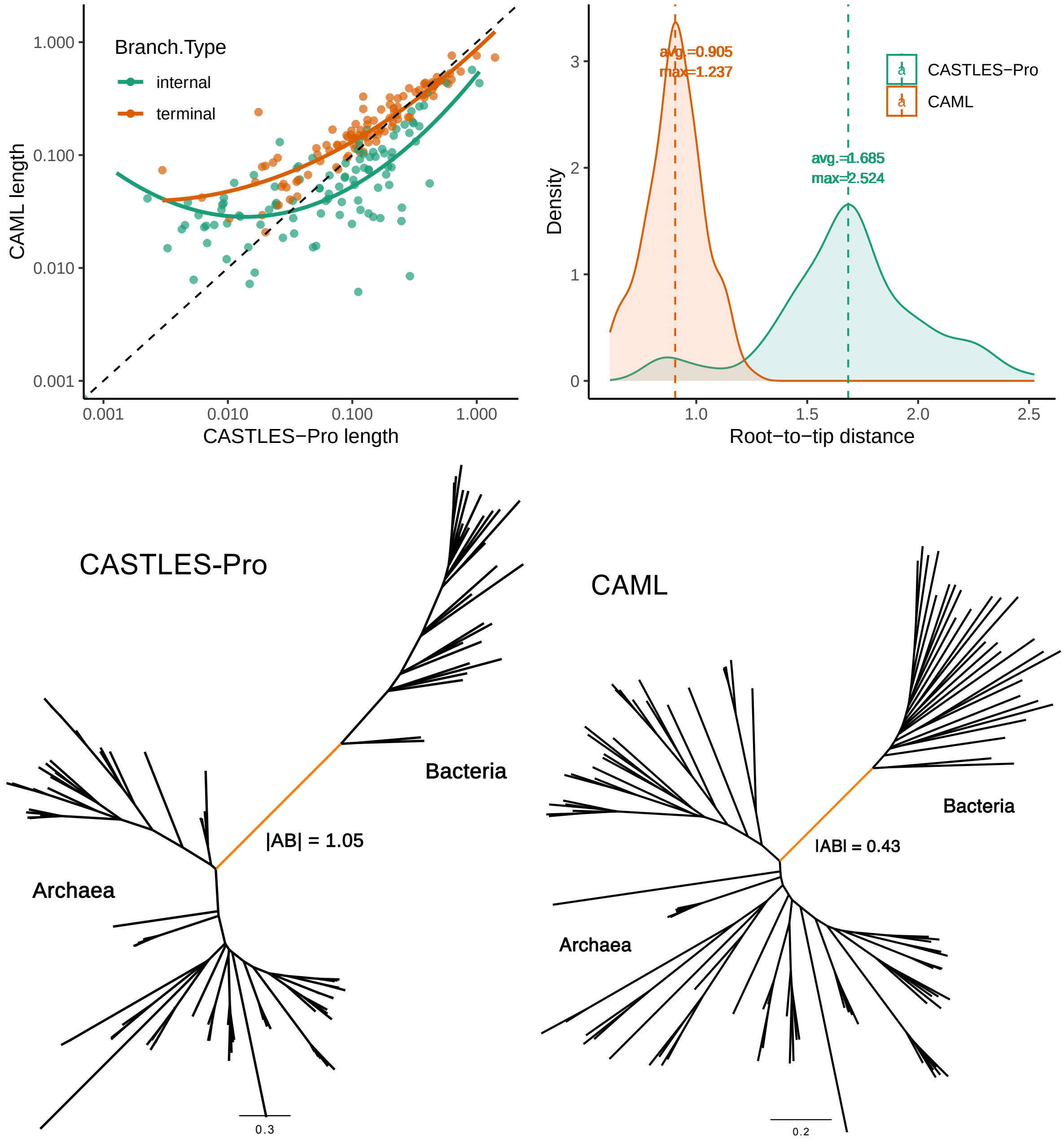

Figure S22: Comparison between branch lengths of CASTLES-Pro and CAML on the bacterial dataset with non-ribosomal genes from Petitjean et al. (2015). The number of species is 108 and the number of genes is 38. Both methods draw branch lengths on an ASTRAL tree topology. The total alignment length is 6,534bp. The branch colored in orange is the AB branch.

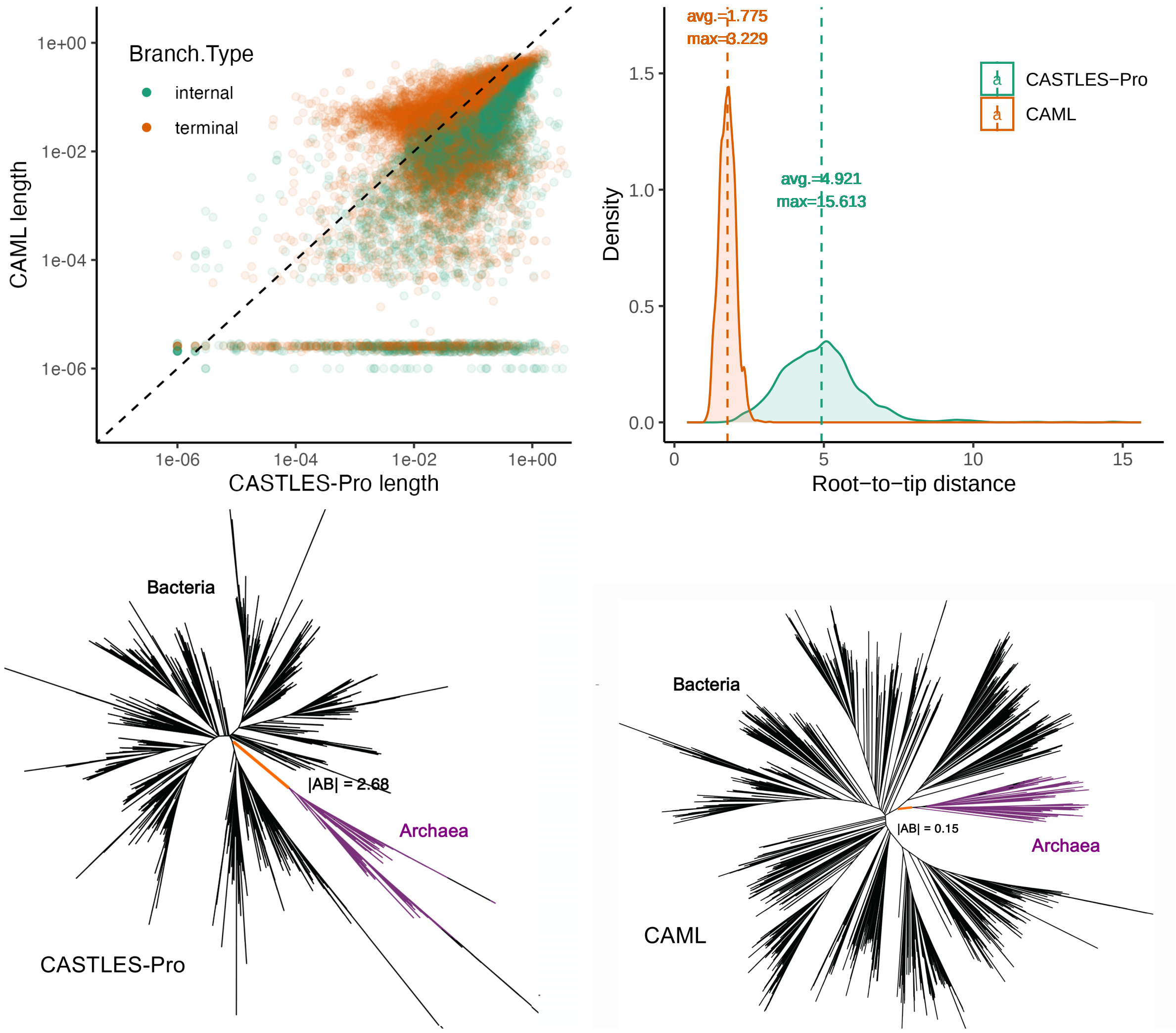

Figure S23: Comparison between branch lengths of CASTLES-Pro and CAML on the Web of Life (WoL) bacterial dataset from Zhu et al. (2019). The number of species is 10,575 and the number of marker genes is 381. The original study had estimated an ASTRAL tree topology and furnished that with concatenation branch lengths, using a concatenated alignment of size 38kbp. This alignment was created by selecting 100 random sites from each gene sequence, and was 5X shorter than the full-length alignment that had 192k sites in total. We draw branch lengths on the same tree topology using CASTLES-Pro. The long tail of branches with 1e-6 or 2e-6 length in the concatenation tree correspond to no-event branches. The branch colored in orange is the AB branch.

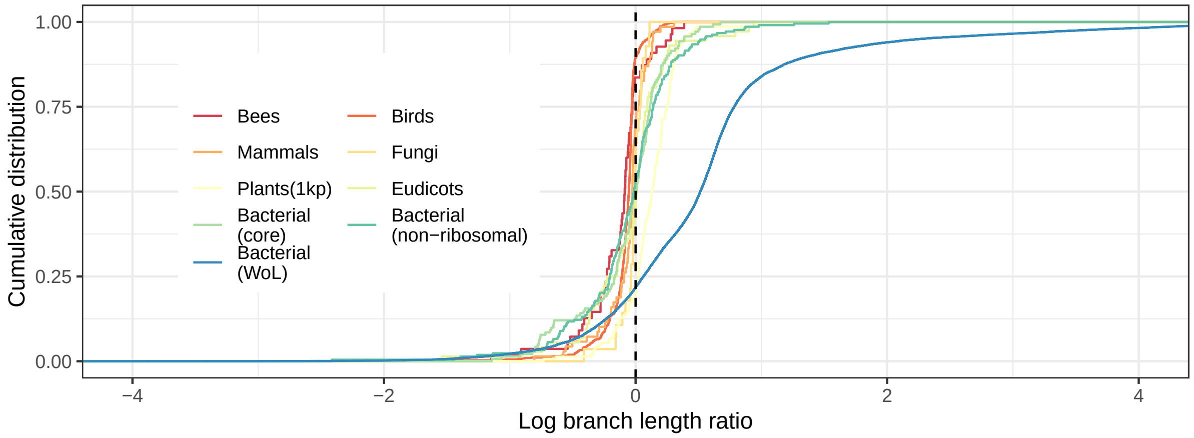

Figure S24: Cumulative distribution of the log ratio between the branch lengths produced by CASTLES-Pro and branch lengths produced by concatenation on nine biological datasets with different sources of gene tree heterogeneity.

Table S7: Runtime and peak memory usage of CASTLES-Pro on the biological datasets. Branch lengths are estimated on a fixed species tree topology. CASTLES-Pro uses multi-copy gene trees for the fungi, plants, and eudicots dataset and single-copy gene trees for the rest of the datasets.

Dataset	# taxa	# genes	time (seconds)	peak memory (GB)
Neoavian birds	363	63,430	2034.64	54.83
Bees (subfamily Nomiinae)	32	853	1.85	0.06
Mammals	37	424	8.60	0.05
Fungi	16	7,180	1.60	0.20
Plants (1kp)	83	9,610	35.92	3.16
Eudicots	40	2,573	26.28	0.63
Bacterial (core genes)	72	49	1.72	0.01
Bacterial (non-ribosomal genes)	108	38	1.89	0.01
Bacterial (WoL)	10,575	381	3180.94	14.48

References

Arasti, S., Tabaghi, P., Tabatabaee, Y., and Mirarab, S. (2024). Branch length transforms using optimal tree metric matching. *bioRxiv*, page 10.1101/2023.11.13.566962. https://www.biorxiv.org/content/10.1101/2023.11.13.566962.

Binet, M., Gascuel, O., Scornavacca, C., P. Douzery, E. J., and Pardi, F. (2016). Fast and accurate branch lengths estimation for phylogenomic trees. *BMC bioinformatics*, 17:1–18.

Bossert, S., Murray, E. A., Pauly, A., Chernyshov, K., Brady, S. G., and Danforth, B. N. (2021). Gene tree estimation error with ultraconserved elements: an empirical study on pseudapis bees. *Systematic Biology*, 70(4):803–821.

Butler, G., Rasmussen, M. D., Lin, M. F., Santos, M. A., Sakthikumar, S., Munro, C. A., Rheinbay, E., Grabherr, M., Forche, A., Reedy, J. L., et al. (2009). Evolution of pathogenicity and sexual reproduction in eight candida genomes. *Nature*, 459(7247):657–662.

Chanderbali, A. S., Jin, L., Xu, Q., Zhang, Y., Zhang, J., Jian, S., Carroll, E., Sankoff, D., Albert, V. A., Howarth, D. G., et al. (2022). Buxus and tetracentron genomes help resolve eudicot genome history. *Nature communications*, 13(1):643.

Cox, C. J., Foster, P. G., Hirt, R. P., Harris, S. R., and Embley, T. M. (2008). The archaebacterial origin of eukaryotes. *Proceedings of the National Academy of Sciences*, 105(51):20356–20361.

Davidson, R., Vachaspati, P., Mirarab, S., and Warnow, T. (2015). Phylogenomic species tree estimation in the presence of incomplete lineage sorting and horizontal gene transfer. *BMC Genomics*, 16(Suppl 10):S1.

Fletcher, W. and Yang, Z. (2009). INDELible: A Flexible Simulator of Biological Sequence Evolution. *Molecular Biology and Evolution*, 26(8):1879–1888.

Gogarten, J. P., Kibak, H., Dittrich, P., Taiz, L., Bowman, E. J., Bowman, B. J., Manolson, M. F., Poole, R. J., Date, T., Oshima, T., et al. (1989). Evolution of the vacuolar h+-atpase: implications for the origin of eukaryotes. *Proceedings of the National Academy of Sciences*, 86(17):6661–6665.

Huelsenbeck, J. P. and Ronquist, F. (2001). MRBAYES: Bayesian inference of phylogenetic trees. *Bioinformatics*, 17(8):754–755. ISBN: 1367-4803 (Print)\r1367-4803 (Linking).

Iwabe, N., Kuma, K.-i., Hasegawa, M., Osawa, S., and Miyata, T. (1989). Evolutionary relationship of archaebacteria, eubacteria, and eukaryotes inferred from phylogenetic trees of duplicated genes. *Proceedings of the National Academy of Sciences*, 86(23):9355–9359.

Johnson, M. G., Pokorny, L., Dodsworth, S., Botigué, L. R., Cowan, R. S., Devault, A., Eiserhardt, W. L., Epitawalage, N., Forest, F., Kim, J. T., et al. (2019). A universal probe set for targeted sequencing of 353 nuclear genes from any flowering plant designed using k-medoids clustering. *Systematic biology*, 68(4):594–606.

Lefort, V., Desper, R., and Gascuel, O. (2015). FastME 2.0: A comprehensive, accurate, and fast distance-based phylogeny inference program. *Molecular Biology and Evolution*, 32(10):2798–2800. ISBN: 1537-1719 (Electronic)\r0737-4038 (Linking).

Mirarab, S., Reaz, R., Bayzid, M. S., Zimmermann, T., Swenson, M. S., and Warnow, T. (2014). ASTRAL: genome-scale coalescent-based species tree estimation. *Bioinformatics*, 30(17):i541–i548.

Moody, E. R., Mahendrarajah, T. A., Dombrowski, N., Clark, J. W., Petitjean, C., Offre, P., Szöllősi, G. J., Spang, A., and Williams, T. A. (2022). An estimate of the deepest branches of the tree of life from ancient vertically evolving genes. *Elife*, 11:e66695.

Petitjean, C., Deschamps, P., López-García, P., and Moreira, D. (2015). Rooting the domain archaea by phylogenomic analysis supports the foundation of the new kingdom proteoarchaeota. *Genome biology and evolution*, 7(1):191–204.

Price, M. N., Dehal, P. S., and Arkin, A. P. (2010). FastTree-2 – Approximately Maximum-Likelihood Trees for Large Alignments. *PLoS ONE*, 5(3):e9490. Publisher: Public Library of Science.

Robinson, D. F. and Foulds, L. R. (1981). Comparison of phylogenetic trees. *Math Biosci*, 53(1-2):131–147.

Sayyari, E. and Mirarab, S. (2016). Fast coalescent-based computation of local branch support from quartet frequencies. *Molecular biology and evolution*, 33(7):1654–1668.

Simão, F. A., Waterhouse, R. M., Ioannidis, P., Kriventseva, E. V., and Zdobnov, E. M. (2015). BUSCO: assessing genome assembly and annotation completeness with single-copy orthologs. *Bioinformatics*, 31(19):3210–3212.

Song, S., Liu, L., Edwards, S. V., et al. (2012). Resolving conflict in eutherian mammal phylogeny using phylogenomics and the multispecies coalescent model. *Proc Natl Acad Sci U S A*, 109(37):14942–14947.

Stamatakis, A. (2014). RAxML version 8: a tool for phylogenetic analysis and post-analysis of large phylogenies. *Bioinformatics*, 30(9):1312–1313.

Stiller, J., Feng, S., Chowdhury, A.-A., Rivas-González, I., Duchêne, D. A., Fang, Q., Deng, Y., Kozlov, A., Stamatakis, A., Claramunt, S., et al. (2024). Complexity of avian evolution revealed by family-level genomes. *Nature*, 629(8013):851–860.

Sukumaran, J. and Holder, M. T. (2010). DendroPy: a Python library for phylogenetic computing. *Bioinformatics*, 26(12):1569–1571. Publisher: Department of Ecology and Evolutionary Biology, University of Kansas, Lawrence, USA. jeet@ku.edu.

Tabatabaee, Y., Zhang, C., Warnow, T., and Mirarab, S. (2023). Phylogenomic branch length estimation using quartets. *Bioinformatics*, 39(Supplement_1):i185–i193.

Wickett, N. J., Mirarab, S., Nguyen, N., Warnow, T., Carpenter, E., Matasci, N., Ayyampalayam, S., Barker, M. S., Burleigh, J. G., Gitzendanner, M. A., et al. (2014). Phylotranscriptomic analysis of the origin and early diversification of land plants. *Proceedings of the National Academy of Sciences*, 111(45):E4859–E4868.

Williams, T. A., Cox, C. J., Foster, P. G., Szöllősi, G. J., and Embley, T. M. (2020). Phylogenomics provides robust support for a two-domains tree of life. *Nature ecology & evolution*, 4(1):138–147.

Willson, J., Roddur, M. S., Liu, B., Zaharias, P., and Warnow, T. (2022). DISCO: species tree inference using multicopy gene family tree decomposition. *Systematic biology*, 71(3):610–629.

Willson, J., Tabatabaee, Y., Liu, B., and Warnow, T. (2023). DISCO+QR: rooting species trees in the presence of GDL and ILS. *Bioinformatics Advances*, 3(1):vbad015.

Zhang, C. and Mirarab, S. (2022). ASTRAL-Pro 2: ultrafast species tree reconstruction from multi-copy gene family trees. *Bioinformatics*, 38(21):4949–4950.

Zhu, Q., Mai, U., Pfeiffer, W., Janssen, S., Asnicar, F., Sanders, J. G., Belda-Ferre, P., Al-Ghalith, G. A., Kopylova, E., McDonald, D., et al. (2019). Phylogenomics of 10,575 genomes reveals evolutionary proximity between domains bacteria and archaea. *Nature communications*, 10(1):5477.
